# Characterization of emergent toxigenic M1_UK_ *Streptococcus pyogenes* and associated sublineages

**DOI:** 10.1099/mgen.0.000994

**Published:** 2023-04-24

**Authors:** Ho Kwong Li, Xiangyun Zhi, Ana Vieira, Harry J. Whitwell, Amelia Schricker, Elita Jauneikaite, Hanqi Li, Ahmed Yosef, Ivan Andrew, Laurence Game, Claire E. Turner, Theresa Lamagni, Juliana Coelho, Shiranee Sriskandan

**Affiliations:** ^1^​ Department of Infectious Disease, Imperial College London, London, UK; ^2^​ MRC Centre for Molecular Bacteriology & Infection (CMBI), Imperial College London, London, UK; ^3^​ National Phenome Centre and Imperial Clinical Phenotyping Centre, Department of Metabolism, Digestion and Reproduction, Imperial College London, London, UK; ^4^​ Department of Metabolism, Digestion and Reproduction, Imperial College London, London, UK; ^5^​ UK Dementia Research Institute, Department of Brain Sciences, Imperial College London, London, UK; ^6^​ NIHR Health Protection Unit in Healthcare-associated Infection and Antimicrobial Resistance, Imperial College London, London, UK; ^7^​ School of Public Health, Imperial College London, London, UK; ^8^​ Genomics Facility, UKRI-MRC London Institute for Medical Sciences (LMS), Imperial College London, London, UK; ^9^​ The Florey Institute, School of Biosciences, University of Sheffield, South Yorkshire, UK; ^10^​ Centre for Infections, UK Health Security Agency, London, UK

**Keywords:** *Streptococcus pyogenes*, superantigen, scarlet fever, genomics, proteome

## Abstract

*
Streptococcus pyogenes* genotype *emm*1 is a successful, globally distributed epidemic clone that is regarded as inherently virulent. An *emm*1 sublineage, M1_UK_, that produces increased levels of SpeA toxin was associated with increased scarlet fever and invasive infections in England in 2015/2016. Defined by 27 SNPs in the core genome, M1_UK_ is now dominant in England. To more fully characterize M1_UK_, we undertook comparative transcriptomic and proteomic analyses of M1_UK_ and contemporary non-M1_UK_ *emm*1 strains (M1_global_). Just seven genes were differentially expressed by M1_UK_ compared with contemporary M1_global_ strains. In addition to *speA*, five genes in the operon that includes glycerol dehydrogenase were upregulated in M1_UK_ (*gldA, mipB/talC, pflD*, and phosphotransferase system IIC and IIB components), while aquaporin (*glpF2*) was downregulated. M1_UK_ strains have a stop codon in *gldA*. Deletion of *gldA* in M1_global_ abrogated glycerol dehydrogenase activity, and recapitulated upregulation of gene expression within the operon that includes *gldA*, consistent with a feedback effect. Phylogenetic analysis identified two intermediate *emm*1 sublineages in England comprising 13/27 (M1_13SNPs_) and 23/27 SNPs (M1_23SNPs_), respectively, that had failed to expand in the population. Proteomic analysis of invasive strains from the four phylogenetic *emm*1 groups highlighted sublineage-specific changes in carbohydrate metabolism, protein synthesis and protein processing; upregulation of SpeA was not observed in chemically defined medium. In rich broth, however, expression of SpeA was upregulated ~10-fold in both M1_23SNPs_ and M1_UK_ sublineages, compared with M1_13SNPs_ and M1_global_. We conclude that stepwise accumulation of SNPs led to the emergence of M1_UK_. While increased expression of SpeA is a key indicator of M1_UK_ and undoubtedly important, M1_UK_ strains have outcompeted M1_23SNPs_ and other *emm* types that produce similar or more superantigen toxin. We speculate that an accumulation of adaptive SNPs has contributed to a wider fitness advantage in M1_UK_ on an inherently successful *emm*1 streptococcal background.

## Data Summary

RNA-seq: all new RNA-seq data are uploaded to the European Nucleotide Archive under project reference PRJEB58303. Genomic data: all genomes listed are available on the European Nucleotide Archive using accession numbers as listed in the appendix. Proteomic data are available as a Microbiology Society FigShare item [1]: https://doi.org/10.6084/m9.figshare.22138172.v1

**Impact Statement**

Although the major *Streptococcus pyogenes* reservoir is in children with pharyngitis and skin infections, *S. pyogenes* can lead to rarer, invasive infections that are rapidly progressive and associated with high mortality and morbidity. *Emm*1 *S. pyogenes* strains are the single most frequent genotype causing invasive infections in high-income countries and are established worldwide as an epidemic clone. The M1_UK_ *S. pyogenes emm*1 sublineage, which is defined by 27 new SNPs in the core genome, and characterized by increased scarlet fever toxin SpeA production, emerged and rose to dominance over a period of 5–6 years since initial recognition, outcompeting other *emm*1 strains in England. Increased dominance of *emm*1 among invasive infections in the winter of 2022/23, on a background of already-increased numbers of *S. pyogenes* infections, points to a key shift in host–pathogen interaction. We hypothesize that a combination of pathogen fitness, virulence and host susceptibility have coalesced to account for the excess of circulating *S. pyogenes* and *emm*1 invasive infections. In this paper we undertake a systems-based evaluation of M1_UK_ in comparison to older non-M1_UK_ *emm*1 strains, and identify a number of pathways that are altered in addition to the previously reported increased SpeA expression. The emergence of a new sublineage within an already virulent clone requires ongoing surveillance, and more detailed investigation of the likely mechanisms leading to increased fitness. The capacity of *S. pyogenes* to cause outbreaks at a national scale highlights a potential need to consider strain-specific public health guidance, underlining the inherent virulence of this exclusively human pathogen.

## Introduction

The modern-day *Streptococcus pyogenes emm*1 genotype emerged in the 1980s and spread globally to become the leading cause of invasive *S. pyogenes* infection throughout the developed world [2, 3]. The lineage expanded following a recombination event that conferred increased expression of the NADase/streptolysin O (*nga/slo*) toxin gene locus, and was associated with specific prophage content, including a prophage encoding the superantigen, streptococcal pyrogenic exotoxin A (SpeA) [2]. More recently, during a period of increased scarlet fever activity in England, a new sublineage of *emm*1 *S. pyogenes* (M1_UK_) was detected and found to have expanded [4]. These M1_UK_ strains were strongly associated with not only sore throats and scarlet fever, but also increases in cases of invasive infection [4]. The earliest M1_UK_ strain detected to date was in a collection of non-invasive infection isolates from London in 2010, while the first invasive strains were detected in England in 2012. By 2016, the M1_UK_ sublineage represented around 80 % of all invasive *emm*1 isolates in England [4]; this rose to 91 % by the end of 2020 [5]. Despite differing from older *emm*1 strains by just 27 core genome SNPs, the new sublineage was characterized by a ten-fold increase in transcription and 9.5-fold increase in median production of the superantigen SpeA. Since 2019, the M1_UK_ lineage has been identified elsewhere in Europe and North America [6–8].

*Emm*1 strains are the single most dominant cause of invasive *S. pyogenes* infection in developed countries. In this work, we set out to characterize the wider phenotype of the new sublineage M1_UK_, and to compare M1_UK_ strains with minor sublineages that appeared briefly as intermediates, although did not expand to the extent of M1_UK_. We also examined natural mutants of M1_UK_ and the minor sublineages that provide insight into the cost–benefit balance of the changes in this new highly successful group of *S. pyogenes* M1T1 strains.

## Methods

### Bacterial strains

*S. pyogenes* strains used are outlined in Tables S1 and S2, available in the online version of this article; strains stored in 20 % glycerol were streaked onto Columbia blood agar (CBA) prior to broth culture. *S. pyogenes* were cultured in Todd Hewitt Broth (THB; Oxoid) or chemically defined medium (CDM) comprising iron, phosphate, magnesium, manganese, sodium acetate, calcium, sodium bicarbonate, l-cysteine, bases, vitamins and amino acids, with different carbon sources (Table S3) at 37 °C in 5 % CO_2_.

### RNA sequencing

RNA was extracted from four different *S. pyogenes* strains from each lineage (Table S1), cultured in THB for 6 h corresponding to late-log growth phase using methods as previously described [4]. RNA sequencing (RNA-seq) of M1_global_ and M1_UK_ RNA was undertaken by Novogene and by the MRC London Institute of Medical Sciences (LMS). Data (deposited in project PRJEB58303) were analysed according to published guidelines [9]. Briefly, read quality was accessed using FastQC (https://www.bioinformatics.babraham.ac.uk/projects/fastqc/), filtered and trimmed using trimmomatic [10], and mapped against the MGAS5005 (CP000017) reference genome using bowtie2 [11] with the highest sensitivity options. The resulting alignments were converted to sorted BAM files using vcftools [12]. Initial visualizations of the sequencing mapping were performed using the Integrative Genomics Viewer (IGV) [13] including confirmation of *gldA* disruption. The mapped RNA-seq reads were then transformed into a fragment count per gene per sample using the HT-seq [14] package. Exploratory data analysis (principal component analysis and heatmap of sample-to-sample distances) of the RNA-seq data was implemented and plotted using the DESeq2 package [15]. Differential expression analysis in each dataset was performed using three different R packages (DESeq2 [15], EdgeR [16] and limma (https://bioconductor.riken.jp/packages/3.0/bioc/html/limma.html)) with a log_2_fold change of 0.5 and *P*-adj <0.05 for M1_global_ vs. M1_UK_, and a log_2_fold change of 1 and *P*-adj <0.05 for M1_H1488∆gldA_ vs. M1_H1488._ Only genes differentially expressed (DE) in at least two of the three softwares used were considered as DE genes and used in analysis. Prophage regions were predicted using phaster [17], and curated by visual assessment and blast alignment.

### Gene transcription studies

Specific transcript abundance was evaluated by real time quantitative reverse transcription PCR (qRT-PCR) using a plasmid standard for each gene and compared with the housekeeping gene *proS*. For the *gldA* operon plasmid standard, single amplicons were amplified to create a single linear insert (*proS-gldA-mipB-pflD*-PTS subunit IIC) that was TA-cloned into plasmid pCR2.1. For *glpF2* and *speA,* the plasmid standard comprised just *glpF2* and *proS*, or *speA* and *proS* respectively. cDNA synthesis from *S. pyogenes* RNA was undertaken as previously reported prior to RT-PCR [4]; primers are listed in Table S4. Comparisons were subject to analysis in GraphPad Prism v9. Non-parametric (Mann Whitney *U*) or *t*-tests were used; *P*<0.05 was considered significant.

### Genetic manipulation

The gene encoding *gldA* was mutated by allelic replacement using the suicide vector pUCMUT. A 541 bp fragment upstream of the *gldA* gene was amplified (forward primer: 5′*-*AGC*GAATTC*TCGCCCAAGATTACGAAGG-3′, reverse primer: 5′-GG*GGTACC*CGTTGAACTCCTTTATCTGTGATT-3′) incorporating 5′ *Eco*RI and 3′ *Kpn*I restriction sites (shown in italics), and cloned into the suicide vector pUCMUT to produce vector pUCMUT_gldAUP_. A 532 bp fragment downstream of the *gldA* gene was amplified (forward primer: 5′-AA*CTGCAG*CTATTGCAGAGCTGGTGCT-3′, reverse primer: 5′ -ACGC*GTCGAC*CGAGTCGATAGGCTAACC-3′) incorporating 5′ *Pst*I and 3′ *Sal*I restriction sites (shown in italics) and cloned into *Pst*I/*Sal*I digested pUCMUT_gldAUP_ to create pUCMUTgldA_KO_. The construct was introduced into *S. pyogenes* M1_global_ strains H1488 (M1_H1488_) and BHS162 (M1_BHS162_) by electroporation and crossed into the chromosome by homologous recombination. Transformants were selected using kanamycin (400 µg ml^−1^). Successful disruption of the *gldA* gene and insertion of the kanamycin resistance cassette was confirmed by PCR, DNA sequencing and whole genome sequencing of mutated strains M1_H1488ΔgldA_ and M1_BHS162ΔgldA_ (isolate identifiers H1589 and H2151 respectively).

### GldA activity assay

Cell-free extracts were prepared from bacteria cultured overnight in chemically defined medium containing either 0.5 % glucose or 0.5 % glycerol to an *A*_600_ of 0.6–0.7 (or as close to this as feasible). Bacteria were washed, centrifuged and kept on ice for 1 h within an anaerobic jar, then suspended in 10 mM Tris buffer, pH 9. Cells were disrupted by agitation in three 60 s bursts with 0.1 mm glass beads. Beads were allowed to settle, and the supernatant fluid was centrifuged for 30 s at 14 000 ***g***. GldA results in conversion of glycerol+NAD to dihydroxyacetone+NADH+H^+^. GldA activity was derived from the increase in absorbance at 340 nm resulting from the reduction of NAD; one unit reduces one micromole of NAD per minute at 25 °C and pH 10.0 under the specific conditions used [18].

### Phylogenetic analysis

*Emm*1 genomes used in phylogenetic analysis were from the UK and are listed in the Supplementary Information. These comprise sequenced non-invasive *emm*1 isolates (*n*=139) [4]; sequenced invasive *emm*1 isolates (*n*=40) from two studies [4, 19]; 64 invasive *emm*1 isolates from the British Society for Antimicrobial Chemotherapy (BSAC) collection [20]; and 23 *emm*1 isolates from a hospital outbreak study [21]. Two new *emm*1 genomes were sequenced from an additional outbreak and are available from the European nucleotide archive (Project PRJEB36425: ERS4267588 and ERS4267589). Raw reads were trimmed using trimmomatic version 0.36 [10] with default parameters. SNP calling was performed by mapping trimmed reads to the complete *emm*1.0 MGAS5005 (CP000017) reference genome using Snippy v4.6.0 (https://github.com/tseemann/snippy), with a minimum coverage of 10, minimum fraction of 0.9 and minimum vcf variant call quality of 100. Gubbins v2.4.1 [22] was used to identify and remove recombinant regions from the resulting full genome alignment file. A maximum likelihood phylogeny was created from core SNPs using the general time-reversible (GTR) model of nucleotide substitution with the gamma distributed rate heterogeneity implemented in FastTree v2.1.10–4 [23] Phylogenetic trees were visualized using FigTree v1.4.2 (http://tree.bio.ed.ac.uk/software/figtree/) and Microreact (https://microreact.org/showcase) and edited using INKSCAPE (https://inkscape.org/pt/).

### SpeA production

In preliminary work SpeA production by 135 non-invasive strains previously evaluated by transcription [4] was evaluated. SpeA production by 40 invasive *S. pyogenes* isolates was undertaken using cell-free culture supernatants from isolates cultured in THB for 6 h (overnight when testing *n*=135 non-invasive isolates), concentrated 5× using Amicon filters, western blotting using a rabbit polyclonal antibody to rSpeA and comparison with standard concentrations of rSpeA expressed from *Escherichia coli* as previously reported [4]. Supernatants with undetectable levels of SpeA were assigned an arbitrary value of half that of the lowest concentration detectable (usually 12.5 ng ml^−1^).

### Proteomics

In pilot studies (Experiment 1), five strains were randomly selected from each of M1_UK_ or M1_global_, cultured in 50 ml CDM to an *A*_600_ of 1.2–1.4 for 6 h at 37 °C with 5 % CO_2_, and then cytosolic, cell wall and supernatant fractions were prepared for proteomic analysis. For proteomic analysis of sublineages (Experiment 2, cytosolic fractions only), strains were randomly selected from five phylogenetic branches within each lineage (five strains per sublineage, four sublineages in total). The supernatant fraction was removed, syringe filtered (Minisart 0.2 µm filter; Sartorius) and proteins precipitated overnight at 4 °C using 10 % trichloroacetic acid precipitation. Cell-wall proteins were extracted from the bacterial pellet using 1 ml of 30 % raffinose, centrifugation at 10 000 r.p.m. for 5 min, followed by resuspending the pellet in 1 ml of cell wall extraction buffer (960 µl 30 % raffinose, 10 µl of 1 M Tris-HCl pH 8, 10 µl of 10 kU ml^–1^ mutanolysin, 10 µl of 100 mg ml^−1^ lysozyme and 10 µl protease inhibitor cocktail III; Avantor VWR), followed by incubation at 37 °C for 3 h with occasional turning, and then aspiration of cell wall extract supernatant after centrifugation at 13 000 r.p.m. for 10 min. The residual cytosolic fraction was further mechanically lysed via bead beating for three cycles for 45 s (Lysing Matrix B from MP Bio). The samples of each cellular fraction then underwent centrifugal concentration using 3 kDa filters and buffer was exchanged (Amicon Ultra-15; Millipore) with 50 mM Tris buffer at pH 8. The samples were then submitted to the Proteomics Facility of the National Phenome Centre (London, UK) for LC mass spectrometry (MS). Precipitated samples were dissolved in 8 M urea and 100 mM ammonium bicarbonate (AmBic) by sonicating for 10 min in a water bath. Total protein was determined in all samples by a protein assay (Protein Assay II; BioRad) according to the manufacturer’s instructions. In total, 20 µg of protein was digested by the addition of 40 mM chloracetamide, 10 mM TCEP (Bondbreaker; ThermoScientific) and 0.2 µg trypsin in 100 mM AmBic. Proteins in 8 M urea were diluted to 1 M urea prior to the addition of trypsin and all samples left overnight at 37 °C. Desalting was performed by acidifying samples to 0.5 % trifluroacetic acid (TFA) and adding them to a pre-equilibrated uElution HLB desalting plate (Waters), washing (3×100 µl) with 0.5 % TFA and eluting with 80 % acetonitrile (3×50 µl). All washes were drawn through the plate under vacuum. Desalted peptides were dried completely at 45 °C in a vacuum-centrifuge.

For MS analysis, proteins were dissolved in 0.1 % formic acid by sonicating in a water bath for 10 min. In total, 0.5 µg of peptides was separated over 90 min over a gradient of 10–40 % acetonitrile at 0.5 µl min^−1^ (0.1 % formic acid) (M-Class UHPLC; Waters) by trapping (nanoE MZ Sym 5 µm 20 mm; Waters) and eluting across a 20 cm C18 column (nanoE MZ HSS T3 1.8 µm 75 µm×200 mm; Waters) and analysed by high definition mass spectrometry (HDMSe) (Synapt G2S; Waters). Data were acquired over 50–2000*mz* for both low and high energy (switching every 1 s) in positive resolution mode. Lock mass (Leu-enkephalin) was acquired every 60 s. For fragmentation, a ramped collision energy was used between 19 and 45 eV. Data were searched and processed using Progenesis QI for Proteomics (FigShare) [24].

DE proteins with a fold change threshold of 1.5 (*P* value threshold 0.05) were visualized on volcano plots. Data comparisons are provided in the accompanying supporting Excel file [24]. Enrichment analysis and protein-protein interactions were performed using STRING (https://string-db.org/), a database able to predict direct (physical) and indirect (functional) associations based on collected data across a range of experimental and *in silico* protein interactions. Proteins with a percentage identity >90 % and a ‘combined interaction score’ >0.7 were used to create a protein network in which the interaction between two proteins was inferred based on the information available in the STRING database and colour coded accordingly.

## Results

### Transcriptome of M1_UK_ *S. pyogenes*

When comparing four TH broth-cultured M1_UK_ and four M1_global_ *S. pyogenes *, significant differential expression of just seven genes was observed (Table 1). As expected, transcription of *speA* was upregulated ~5-fold in M1_UK_ strains compared with other M1_global_ strains; ~10-fold increased *speA* transcription by M1_UK_ has previously been confirmed by qRT-PCR of RNA from 135 *emm*1 isolates [4]. Unexpectedly, transcription of *glpF2*, a putative aquaporin (identified as Spy1573 in *emm*1 reference strain MGAS5005), was 5-fold downregulated in M1_UK_ strains. Bioinformatic analysis of the *S. pyogenes* aquaporin gene *glpF2* demonstrated similarity to the *glpF3* family of *Lactobacillus plantarum* reported to be associated with both glycerol and water, but also dihydroxyacetone (DHA) transport [25].

**Table 1.** Differentially expressed genes comparing four M1_UK_ and four M1_global_ strains

Gene ID	Gene name	Description	Average log_2_fold change	Average *P*_adj_	Strand
M5005_Spy0996	*speA2*	Enterotoxin	2.361	3.731E-09	+
M5005_Spy1573	*glpF2*	Glycerol uptake facilitator protein	−2.423	5.143E-09	−
M5005_Spy1741	*gldA*	Glycerol dehydrogenase	1.024	0.0006	−
M5005_Spy1742	*mipB*	Transaldolase	1.043	0.0002	−
M5005_Spy1743	*pflD*	Formate acetyltransferase	0.963	0.0007	−
M5005_Spy1744	na	PTS system, cellobiose-specific IIC component	0.653	0.0061	−
M5005_Spy1745	na	PTS system, cellobiose-specific IIB component	0.749	0.0204	−

The remaining five DE transcripts that were upregulated in M1_UK_ represented consecutive ORFs in an apparent operon that includes glycerol dehydrogenase (*gldA*), pyruvate formate lyase (*pflD*) and a transaldolase-like protein (*talC* or *mipB*) as well as phosphotransferase (PTS) system IIC and IIB component genes, annotated as cellobiose-specific.

### GldA operon

**Fig. 1.**
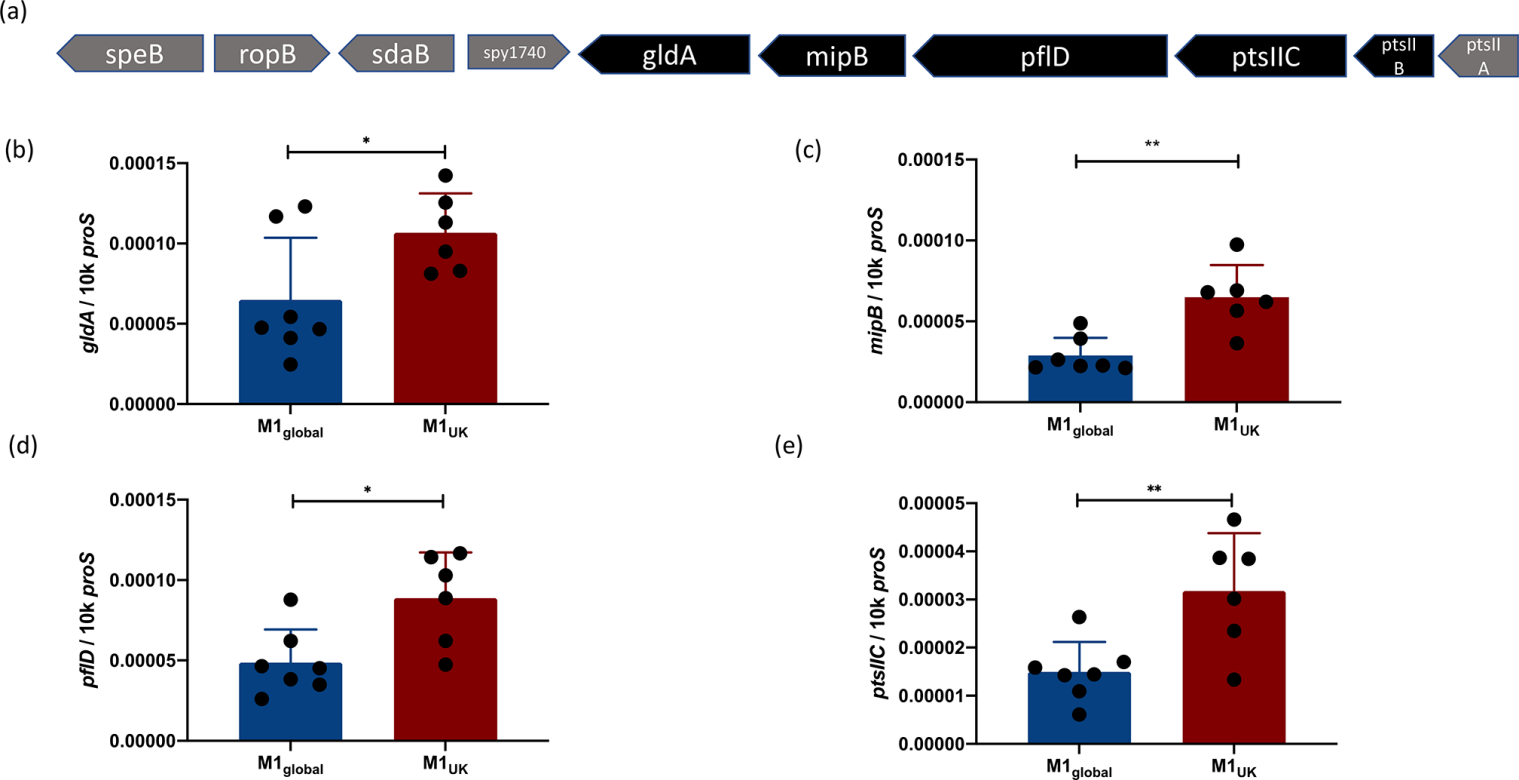
The genes within the *pflD-mipB-gldA* operon are upregulated in M1_UK_. Five adjacent genes were found to be upregulated in M1_UK_ compared to M1_global_ (**a**). Genes upregulated in RNA-seq are shown in black and include two components of a PTS annotated as PTS system (cellobiose) subunits IIC and IIB. qRT-PCR using RNA from M1_global_ (*n*=7) and M1_UK_ (*n*=6) strains indicating transcription of *gldA* (**b**); *mipB*, also known as *talC* (**c**); *pflD* (**d**); and PTS subunit IIC (**e**). Data points (black dots) represent different strains that were each tested in technical triplicates and expressed as copies per 10000 copies of *proS*. Error bars show sd of the mean.**P<0.01 using an unpaired *t*-test; *P<0.05.

A single SNP in the glycerol dehydrogenase gene *gldA* among all M1_UK_ strains is known to introduce a premature stop codon at position 175 of the 362 residue enzyme and is predicted to result in a truncated protein with abrogated enzyme activity [4]. The gene *gldA* is the final ORF in the sequence of genes that was found to be differentially expressed (Fig. 1a). Differential expression of genes comprising the apparent operon was confirmed using qRT-PCR (Fig. 1b–e). Transcription of the aquaporin gene was evaluated in three strains from each lineage, and although non-significant, there was a 2-fold reduction in transcription in M1_UK_ (Fig. S1).

**Fig. 2.**
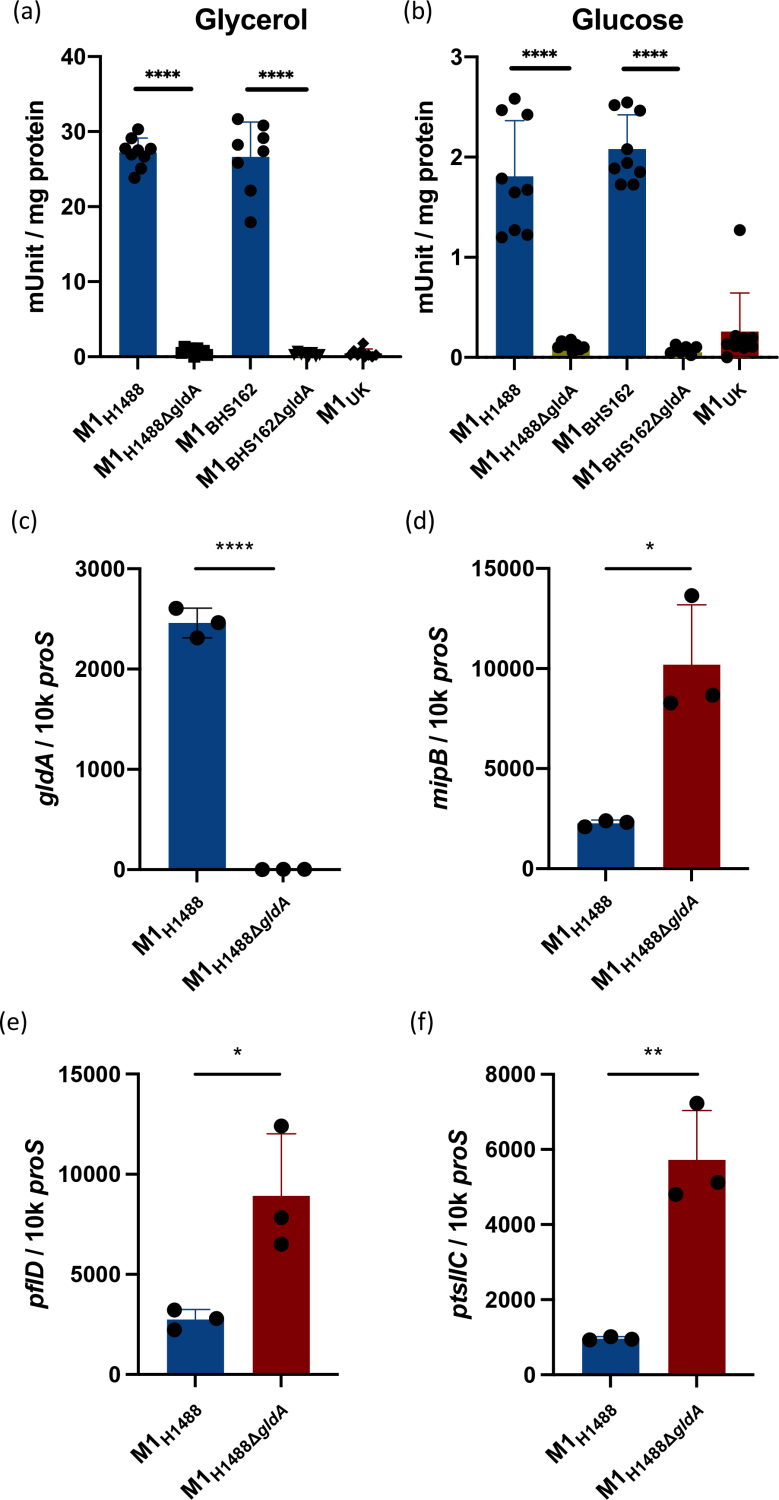
Loss of GldA function is associated with upregulated expression of adjacent genes. Glycerol dehydrogenase activity in *S. pyogenes* M1_global_ is abrogated following inactivation of the *gldA* gene, to the level observed in M1_UK_. Activity in the parent strain was greatest when cultured in the presence of glycerol (**a**) and less in glucose (**b**). Data show eight or nine individual reactions for each strain. Deletion of the *gldA* gene resulted in markedly reduced transcription of *gldA* (**c**), and upregulated transcription of *mipB* (**d**), *pflD* (**e**) and PTS component IIC (**f**). Data show three biological replicates per strain. Error bars show sd of the mean. *P<0.05; **P<0.01; ****P<0.0001 using a Mann–Whitney U test (**a, b**) and unpaired *t*-test (**c–f**).

We hypothesized that the loss of GldA enzyme activity may in some way feedback on transcription of the adjacent PTS subunit EII genes, as well as *mipB* and *pflD*. To determine the impact of isolated loss of GldA function in *S. pyogenes *, *gldA* was disrupted through allelic replacement in M1_global_ strain M1_H1488_ to create M1_H1488ΔgldA_. A GldA enzyme activity assay was undertaken, in the presence of glycerol and glucose, to confirm that enzyme function was present in the parent strain, but abrogated in the mutant (Fig. 2a, b); this was replicated using a second pair of isogenic M1_global_ strains (M1_BHS162_ and M1_BHS162ΔgldA_). By comparison, M1_UK_ strain BHS581 demonstrated barely detectable GldA activity, similar to the knockouts. RNA from M1_H1488_ and the isogenic M1_H1488ΔgldA_ was subject to RNA-seq to compare the wider transcriptome of *S. pyogenes* in the absence of a functional *gldA* gene. There were almost no changes in the transcriptome except in the genes of the putative *pflD–mipB–gldA* operon; deletion of *gldA* abrogated transcription of *gldA* as expected, but was associated with a clear increase in transcription of *pflD*, *mipB* and the adjacent PTS system cellobiose-specific IIC genes. (Table 2). Upregulation of two genes adjacent to one another, Spy0123 and Spy0124 (including *sloR*), was also observed.

**Table 2.** RNA-seq comparison of *gldA*-mutant *S. pyogenes* and parent strain

Gene ID	Gene name	Description	Average log_2_ fold change*	Average *P*_adj_	Strand
M5005_Spy0008	*divIC*	Cell division protein	−1.003	0.032	+
M5005_Spy0123	na	Translation initiation inhibitor	1.152	0.016	+
M5005_Spy0124	*sloR*	Transcriptional regulator	1.250	0.0009	
M5005_Spy1166	na	Hypothetical protein	−1.094	0.0001	−
M5005_Spy1258	na	Putative cytosolic protein	−1.361	0.019	−
M5005_Spy1541	na	Hypothetical protein	−1.030	0.018	−
M5005_Spy1741	*gldA*	Glycerol dehydrogenase	−9.212	1.111E-05	−
M5005_Spy1742	*mipB*	Transaldolase	1.367	1.91E-03	
M5005_Spy1743	*pflD*	Formate acetyltransferase	1.394	0.0019	
M5005_Spy1744	na	PTS system cellobiose-specific IIC	1.036	0.0036	−

*Comparison is made between M1_H1488ΔgldA_ and parent strain M1_H1488_; only genes differentially expressed by at least log_2_ fold value of 1.0 are shown, *P*<0.05. Genes from the same predicted operon are shaded in grey.

Significant downregulation of *gldA* transcription, and upregulation of the adjacent genes was confirmed by qRT-PCR (Fig. 2c–f). Taken together, the data suggested that loss of GldA activity led to upregulation of the entire operon that is concerned with metabolism of DHA, fructose 1,6 phosphate and pyruvate. *S. pyogenes* has been reported to use a number of carbon sources; however, under conditions where *emm1 S. pyogenes* grew well in CDM supplemented with glucose, we were unable to demonstrate any growth in CDM supplemented with glycerol alone, consistent with other reports [26] (data not shown). Informatic analysis of publicly available genomes from a range of bacterial species demonstrated remarkable conservation of the genes and organization of this region in all members of the *Streptococcaciae* compared with other species (Fig. S2).

### Intermediate sublineages of *emm*1 *S. pyogenes*

**Fig. 3.**
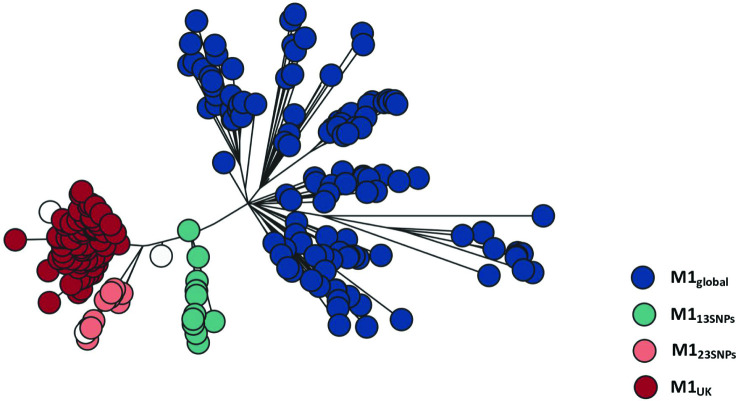
Phylogeny of M1_UK_, M1_global_ and two intermediate sublineages. Maximum likelihood phylogenetic tree reconstructed from core SNPs (without recombination regions) of 269 invasive and non-invasive *emm*1 *S. pyogenes* isolate genomes representative of four main groups (M1_global_, M1_13SNPs_, M1_23SNPs_, M1_UK_). The phylogenetic tree is coloured as described in the key. White bubbles represent isogenic strains from two distinct outbreaks with 26 and 22 SNPs, respectively, and one invasive isolate with 19 SNPs. Isolate whole genome sequences used in the phylogenetic tree are listed in Table S2.

M1_UK_ strains are distinguished from older *emm*1 strains by the presence of 27 SNPs [4] (Table 3). Although a number of additional indels are common in M1_UK_, only the 27 SNPs define the new lineage. When analysing genomes from *S. pyogenes* strains isolated in the UK, we identified small numbers of strains with either 13 or 23 of the 27 SNPs [4]. All *emm*1 sublineages except M1_global_ possessed three SNPs in the transcriptional regulator RofA, but the *gldA* stop codon is present only in strains with 23 or 27 SNPs. (Table 3). We analysed our original non-invasive *S. pyogenes* whole genome sequences alongside other sequenced UK *emm*1 strains (Table S2) and enriched for sublineages by including 10 invasive isolates from each of the following groups: M1_global_, M1_13SNPs_, M1_23SNPs_ and M1_UK_ (Fig. 3). As reported before, the earliest M1_UK_ strain identified was 2010, but the earliest M1_13SNPs_ strain was 2005, from the BSAC collection [20].

**Table 3.** SNPs in sublineages

Position in MGAS5005	Gene locus	Gene	Product	S/NS	Ref	SNP	M1_13 SNPs_	M1_19SNPs_∗	M1_23SNPs_	M1_27 SNPs_†
115 646	M5005_Spy0106	*rofA*	Transcriptional regulator	ns	C	T	T	T	T	T
116 162	M5005_Spy0106	*rofA*	Transcriptional regulator	ns	A	C	C	C	C	C
116 163	M5005_Spy0106	*rofA*	Transcriptional regulator	ns	C	A	A	A	A	A
250 832	M5005_Spy0243		ABC transporter-associated protein	S	T	C	T	T	C	C
513 254	M5005_Spy0525		Galactose-6-phosphate isomerase LacB	ns	G	T	T	T	T	T
528 360	Intergenic		–	–	A	T	A	T	T	T
563 631	M5005_Spy0566	*sagE*	Streptolysin S putative self-immunity protein	ns	G	A	G	G	A	A
613 633	M5005_Spy0609		Phosphoglycerol transferase	ns	T	C	C	C	C	C
626 494	M5005_Spy0623		Methyltransferase	S	G	A	A	A	A	A
661 707	M5005_Spy0656	*trmD*	tRNA (guanine-N (1)-)-methyltransferase	ns	G	A	G	A	A	A
730 823	M5005_Spy0727	*recJ*	ssDNA-specific exonuclease	ns	C	T	T	T	T	T
784 467	M5005_Spy0779		Putative membrane spanning protein	S	T	C	T	T	C	C
819 098	M5005_Spy0825	*murB*	UDP-*N*-acetylenolpyruvoylglucosamine reductase	ns	G	A	G	A	A	A
923 079	M5005_Spy0933		Putative NADH-dependent flavin oxidoreductase	ns	G	A	G	A	A	A
942 633	M5005_Spy0951	*pstB*	Phosphate transport ATP-binding protein	ns	G	T	G	G	G	T
983 438	Intergenic/within ssrA		–	–	G	C	G	G	C	C
1 082 253	M5005_Spy1108	*metK2*	*S*-Adenosylmethionine synthetase	ns	C	T	T	T	T	T
1 238 124	M5005_Spy1282	*msrA*	Peptide methionine sulphoxide reductase	ns	G	A	A	A	A	A
1 238 673	M5005_Spy1283	*tlpA*	Thiol:disulphide interchange protein	ns	G	A	A	A	A	A
1 251 193	M5005_Spy1293		Hypothetical protein	ns	G	A	G	G	G	A
1 373 176	M5005_Spy1400		PTS system, galactose-specific IIB component	ns	C	A	C	C	C	A
1 407 497	M5005_Spy1439		Portal protein	ns	C	T	T	T	T	T
1 446 116	M5005_Spy1490		3-Oxoacyl-[acyl-carrier protein] reductase	S	C	T	T	T	T	T
1 535 209	Intergenic		–	–	A	G	A	A	A	G
1 702 540	M5005_Spy1714	*gldA*	Glycerol dehydrogenase	STOP	C	T	C	T	T	T
1 734 749	M5005_Spy1772		Glutamate formimidoyltransferase	ns	G	A	A	A	A	A
1 828 734	M5005_Spy1860		Putative membrane spanning protein	ns	G	A	G	A	A	A

*Single strain with 19 of the 27 SNPs that characterize M1_UK_ (not a sublineage).

†Lineage with 27 SNPs is equivalent to M1_UK_.

### SpeA expression by sublineages

Previous comparison had demonstrated ~10-fold greater *speA* gene transcription by non-invasive M1_UK_ isolates compared to non-invasive M1_global_ strains [4]; we first established that SpeA protein expression was similarly elevated in the same large panel of non-invasive isolates (Fig. S3). There was an indication that SpeA production was not increased in a small number of strains from intermediate lineages. To better understand the impact of the step-wise changes in SNP content, we examined *speA* gene transcription and protein production in a new set of strains. To include sufficient numbers of intermediate sublineage isolates, we used 40 strains from a larger national collection of invasive *emm*1 *S. pyogenes* that had been submitted to the reference laboratory and were previously sequenced [4, 19].

**Fig. 4.**
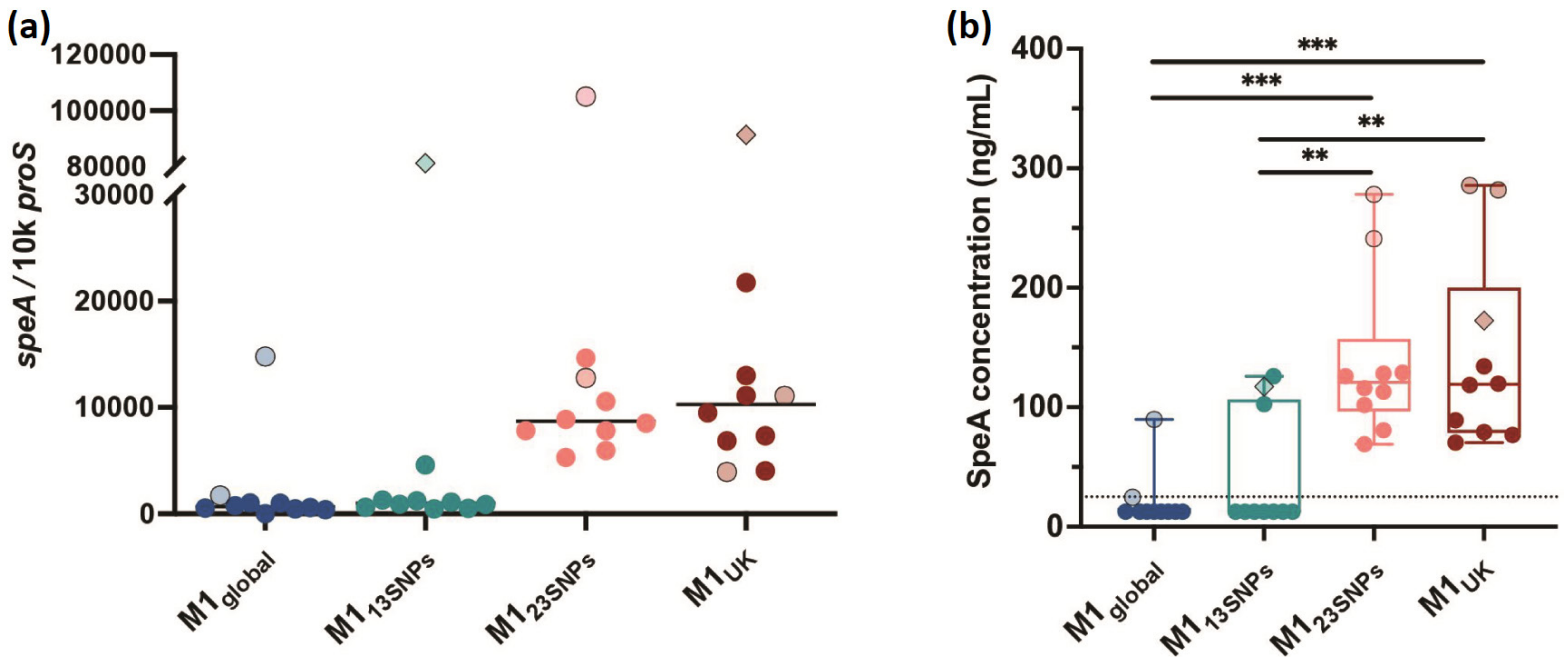
SpeA expression is increased in M1_23SNPs_ and M1_UK_ sublineages. (**a**) Transcription of *speA* using 10 isolates from each sublineage is shown (total *n*=40). Each dot represents a single isolate (measured in triplicate) with light shading/fine black outline indicating the presence of a *covRS* mutation. Two isolates possessed both *covRS* and *rgg4* mutations (diamond shapes). Solid line represents the median. There was no statistically significant difference between the sublineages in *speA* transcription, largely related to the outlying *covRS* mutants in each sublineage. Excluding isolates with *covRS* mutations a difference was observed between M1_global_ and either M1_23SNPs_ or M1_UK_ (*P*<0.0001), and a difference between between M1_13SNPs_ and either M1_23SNPs_ or M1_UK_ (*P*=0.0002). Median fold difference M1_UK_ versus M1_global_ was 15.2 including all strains tested. (**b**) SpeA protein production by 40 invasive isolates (10 in each sublineage) cultured for 6 h in THB. Limit of detection was 25 ng ml^−1^. Isolates with undetectable SpeA were assigned a value of 12.5 ng ml^−1^. Median fold difference M1_UK_ versus M1_global_ was 9.5 including all strains tested. Box plot shows median and range. A multiple comparisons test was made using one-way ANOVA (Tukey’s): **P<0.01; ***P<0.001.

Transcription of *speA* was low in all M1_global_ and M1_13SNPs_ strains, except for the occasional strain with a mutation in *covRS*, a two-component system regulator known to suppress virulence factors, but which can undergo mutation to confer a more invasive phenotype in *emm*1 and other *S. pyogenes* strains. In contrast, transcription of *speA* was high in all invasive strains with 23 or 27 SNPs (Fig. 4a). Likewise, SpeA protein production differed markedly between the sublineages; again SpeA production was greatest in all invasive isolates with 23 or 27 SNPs and was hard to detect in all M1_global_ and M1_13SNPs_ (Fig. 4b). Indeed, the amount of SpeA produced routinely by M1_UK_ strains was similar to that produced by M1_global_ strains with mutations in *covRS*, a regulator that is known to repress *speA* in *emm*1 [27]. We did not detect a difference in production of other virulence factors such as SpyCEP, SpeB or M protein, in broth culture (data not shown). We concluded that the genetic changes required for basal increased *speA* expression in M1_UK_ resided in M1_23SNPs_ but not M1_13SNPs_.

Isogenic isolates that differed by just single SNPs were available from two outbreak settings. Interestingly, in both settings, a single isolate was identified wherein a single SNP from the 27 SNPs that define M1_UK_ reverted to the wild-type. In one daycare centre outbreak, a non-invasive isolate exhibited only 26 of the 27 SNPs but was otherwise identical to an invasive isolate from the same cluster; in this case, the SNP in *trmD*, a tRNA (guanine-*N* (1)-)-methyltransferase, had reverted to the wild-type. This isolate made as much SpeA as the isolate with 27 SNPs.

In a separate hospital outbreak associated with a fatal case of invasive infection caused by the M1_23SNPs_ sublineage [21], one isolate from a healthcare worker was identical to five other isolates in the cluster, apart from one single SNP. This single SNP represented one of the 23 SNPs but is present in both M1_13SNPs_ and M1_23SNPs_, a phage portal protein (Spy1439). This isolate also produced the same amount of SpeA as the parent M1_23SNPs_ strain, demonstrating the SNPs that were dispensible for increased SpeA expression.

A review of published UK *emm*1 genome sequences [20] identified a single strain with 19 of the 27 SNPs among *emm*1 bloodstream isolates. Unlike the sublineage that possessed 23 SNPs, this M1_19SNPs_ strain did not produce detectable quantities of SpeA, pointing to an influential role for the four SNPs that differentiate M1_19SNPs_ and the M1_23SNPs_ sublineage in SpeA expression. Of these four SNPs, two were synonymous SNPs and unlikely to affect phenotype; one was a non-synonymous SNP in *sagE*; while the final change was an SNP that appeared to be intergenic in annotated *emm*1 *S. pyogenes* genomes but lies within the start of the tmRNA *ssrA* [28] upstream of the phage insertion and start site of *speA* (Spy0996 in MGAS5005). RNA-seq read abundance in this region did not show a difference between M1_global_ and M1_UK_ strains, with the exception of the gene encoding SpeA. Abundance of reads in the ‘paratox’ (Spy0995) gene, which is transcribed on the opposite strand to *speA*, was increased in two of four M1_UK_ strains, but this finding was not consistent.

### Proteomic analysis of *S. pyogenes emm*1 sublineages

**Fig. 5.**
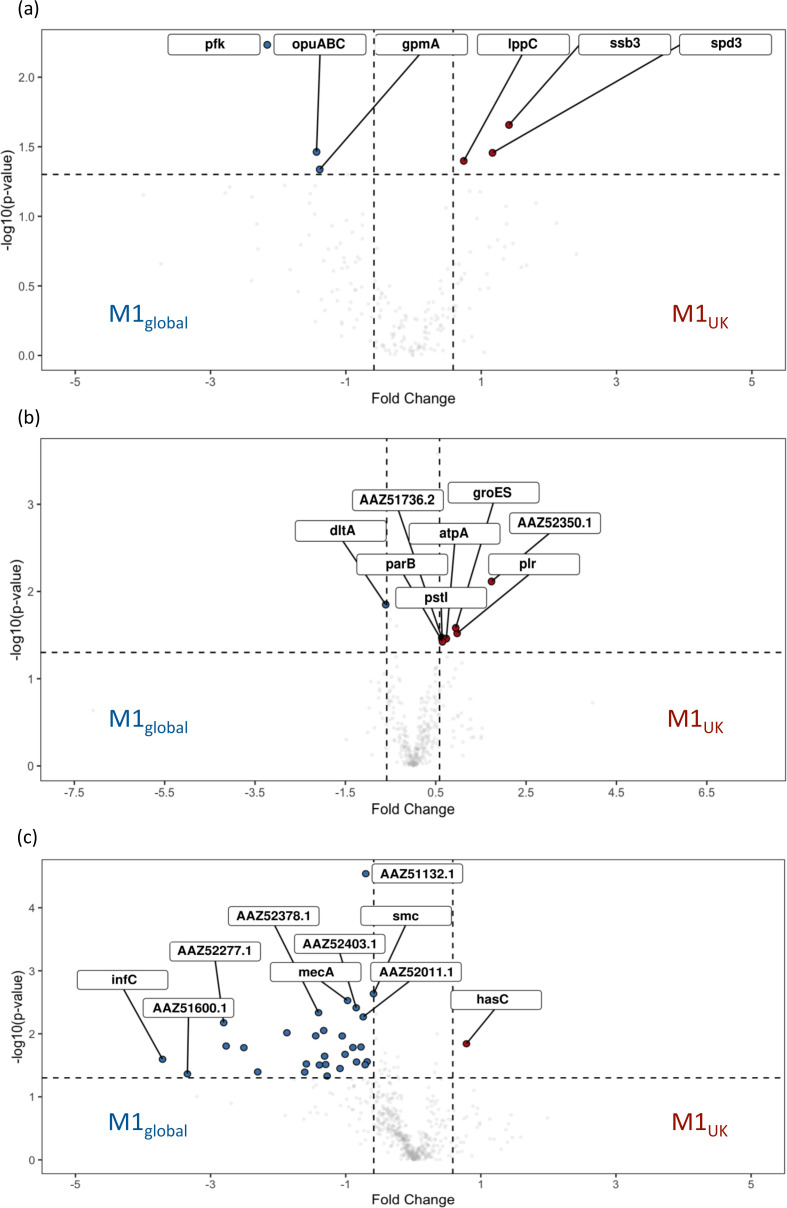
Volcano plots comparing proteins differentially produced by M1_UK_ vs. M1_global_ cultured in CDM. Log_2_ fold change is shown on the *x*-axis. Specific fractions examined were supernatant (**a**), cell wall (**b**) and cytosol (**c**). Proteins increased in M1_UK_ are shown on the right in red. Those increased in M1_global_ are shown on the left in blue. Comparisons are listed in the supporting Excel file (Figshare) [24].

To screen for lineage-specific differences in proteomes, cell wall, cytosolic and supernatant fractions of five randomly selected M1_UK_ isolates were compared with five M1_global_ following culture in CDM. Though SpeA was detected, a significant difference between M1_UK_ and M1_global_ supernatants was not observed when strains were cultured in CDM, in contrast to results (reported above) in THB, pointing to a role for specific culture conditions in induction of SpeA. CDM supernatant from M1_UK_ strains demonstrated increased phage-encoded DNase (Spd3), acid phosphatase (LppC) and a DNA binding protein compared with M1_global_. However, CDM supernatant from M1_global_ demonstrated increased phosphoglycerate mutase and phosphofructokinase, both of which are linked to carbohydrate utilization pathways in *S. pyogenes* (Figs 5a and S4A) [29]. Cell wall fractions demonstrated a small number of proteins that were differentially expressed in M1_UK_ strains. These included a more than 3-fold increase in PrsA2 (Spy1732, AAZ52350.1), which controls protein folding and may operate at the ExPortal [30], and almost 2-fold increases in GAPDH and the 10 kDa chaperonin GroES compared with M1_global_ (Figs 5b and S4B).

In M1_global_ strains, several cytosolic proteins were increased compared to M1_UK_, including those encoded by adjacent genes Spy0438 (*rnc*, ribonuclease III) and Spy0439 (*smc*) as well as *mecA*, coding for an adapter protein and negative regulator of competence; the greatest fold changes were, however, seen in InfC, an Initiation Factor 3, SatD, and a number of proteins linked to protein secretion (SecA), maintenance of ribosomal function and RNA. String analysis highlighted links between phosphoentomutase (DeoB), protein synthesis pathways (GidA) and acid tolerance (SatD) (Figs 5c and S4C).

**Fig. 6.**
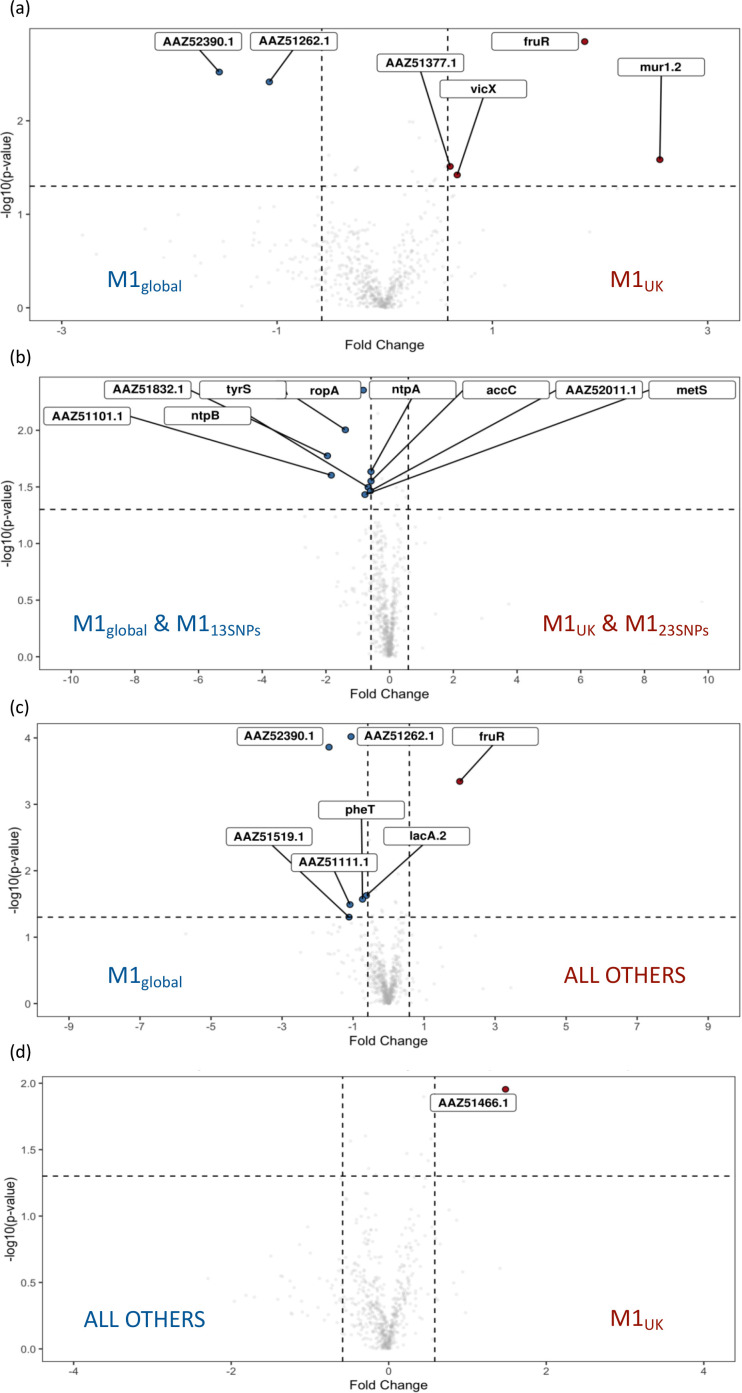
Volcano plots comparing proteins differentially produced when considering different pairings of M1_global_, M1_13SNPs_, M1_23SNPs_ and M1_UK_ cultured in CDM. Log_2_ fold change is shown on the *x*-axis. Cytosolic fractions only were compared as follows: (**a**) M1_UK_ vs. M1_global_, (**b**) [M1_UK_ + M1_23SNPs_] vs. [M1_global_ + M1_13SNPs_], (**c**) all other sublineages vs. M1_global_, and (**d**) M1_UK_ vs. all other sublineages. Comparisons are listed in the supporting Excel file (Figshare) [24].

To screen for differences between all four sublineages (two intermediate and two major sublineages), five isolates from each intermediate sublineage (M1_13SNPs_ and M1_23SNPs_) were randomly selected from appropriate phylogenetic branches as well as five new isolates from each of M1_UK_ and M1_global_. Fresh cytosolic fractions of the four phylogenetic groups (20 strains) were prepared and subject to new proteomic analysis. The data were then analysed by comparing groups in different combinations. When cytosolic preparations from all four sublineages were compared with one another, FruR production was increased in M1_23SNPs_ in comparison to other lineages, and lowest in M1_global_, while a network of ribosomal proteins was increased in M1_13SNPs_ (Fig. S5A). Comparison of cytosolic preparations from new M1_UK_ and M1_global_ strains did not identify the same DE features seen previously; however, a negative regulator of competence, MecA, again was increased in M1_global_ strains although only by 1.3-fold (Fig. S5B). The biggest fold change was a 3.6-fold increase in FruR and 5.87-fold increase in Mur1.2, a potential autolysin (adjacent to a PTS fructose-specific IIABC system and *fruR*), in M1_UK_ isolates (Fig. 6a). As M1_UK_ and M1_23SNPs_ strains had demonstrated comparable SpeA production, we proceeded to determine if there was commonality between these two sublineages by comparing cytosolic proteomes of [M1_UK_ and M1_23SNPs_] with [M1_global_ and M1_13SNPs_]. NtpA and B, a V type ATPase, was increased in [M1_global_ and M1_13SNPs_]. Proteins linked to ligase activity were found to be enriched in string analysis and highest in [M1_global_ and M1_13SNPs_] (Figs 6b and S5C). When considering M1_global_ compared with all other three ‘new’ lineages, carbohydrate metabolism genes were further highlighted, specifically PTS system and disaccharide metabolic processes (Fig. S5D). As observed, FruR was four-fold increased in non-M1_global_ strains, with increased FruA in M1_global_ strains; a similar pattern was seen for LacR and LacA1/LacA2 (Figs 6c and S5D). A glutamate formiminotransferase (MGAS5005_Spy1772) was also increased in M1_global_ strains compared with non-M1_global_. Finally, comparing cytosolic proteins in M1_UK_ with all other lineages, just one protein was clearly relatively increased in M1_UK_, and this was Spy0848 (PpnK), an ATP-NAD kinase (Figs 6d and S5E).

## Discussion

M1_UK_ is now the dominant *S. pyogenes emm*1 lineage in the UK, having expanded during an earlier upsurge in scarlet fever in 2014–2016 [4, 5]. Importantly, *emm*1 strains are inherently virulent and represent the single most frequent *emm* type causing invasive infections in the UK [31, 32]. As such, any change in the *emm*1 lineage that results in increased fitness is of relevance to public health. In this first systematic study to characterize the changes in M1_UK_ and its associated lineages, we have confirmed the *speA* over-expression phenotype, and demonstrated that increased SpeA production is restricted to M1_UK_ and an increasingly rare sublineage M1_23SNPs_. The phenotype is manifest in broth culture but not CDM. M1_UK_ is defined by just 27 SNPs in the core genome, including three SNPs in a stand-alone regulator *rofA* and a stop codon in glycerol dehydrogenase, *gldA*. RNA-seq demonstrated a difference in expression of the operon that includes *gldA* and a PTS system EIIC and B which represents a combined phosphate and sugar transporter, pointing to a potential shift in metabolism in the new lineage. This was accompanied by a sharp reduction in transcripts for the aquaporin gene *glpF2*. Preliminary proteomic analysis of strains by sublineage identified altered carbohydrate pathways related to fructose that may well be important.

Alterations in expression of *gldA, mipB, pflD*, and the adjacently encoded PTS system impact on the glycolytic Embden–Meyerhof–Parnas pathway [29] which, in *S. pyogenes,* relies on the phospho-enolpyruvate PTS system for acquisition of sugars other than glucose, and for transfer of phosphate ions required for carbon catabolite repression and gene regulation [33].

The results indicate that both the premature stop codon in *gldA* present in M1_UK_ and allelic replacement of *gldA* impact glycerol dehydrogenase activity and result in upregulation of the other genes in the operon, *mipB* and *pflD*. These are involved in the glycolytic pathway required for generation and metabolism of pyruvate from glucose; the changes in carbohydrate metabolism are supported by proteomic findings that indicate alterations in fructose pathways. Interestingly, increased transcription in this operon was accompanied by increased transcription of adjacent PTS components IIC and IIB when comparing M1_UK_ with M1_global_, and following experimental deletion of *gldA*. This PTS is annotated as being a cellobiose transporter; systematic experimental disruption of PTS EII systems in *S. pyogenes* has not shown an essential role for these genes, but the precise sugar transported is not known [33].

The role of GldA in *S. pyogenes* has not been experimentally examined previously; GldA is reported to catalyse the conversion of glycerol to DHA under microaerophilic or anaerobic conditions, but it is clear that GldA may also undertake a reverse role, which is to catalyse DHA to glycerol. This may be of importance since an absence of *gldA* activity may lead to a build-up of DHA, which when converted to methylglyoxal can be toxic [34]. Upregulation of the PTS system is of interest since these are recognized to be key players in a phosphorelay process that maintains central carbon catabolite repression of many virulence systems in *S. pyogenes* [33, 35].

The marked ~5-fold downregulation of aquaporin *glpF2* (Spy1573) transcription in M1_UK_ compared to M1_global_ was unexpected but may represent an adaptation to the metabolic changes that have arisen in M1_UK_. There are few reports, if any, relating to *glpF2* in *S. pyogenes* but there is evidence of functional links to the *pflD*-containing operon in enterococci [36]. Notably one of the intergenic SNPs that defines M1_UK_ is 39 bp from the start codon of the Spy1573 gene, though the significance of this is not yet known. Aquaporins are membrane proteins that function as channels for water and other uncharged solutes in all forms of life. While mostly considered as channels for water or glycerol in bacteria, potentially important to osmoregulation, aquaporins also can function as a channel for DHA. Indeed, there are similarities between *glpF2* of streptococci and *glpF3* of *Lactobacillus plantarum* that points to a possibility for action as a channel for DHA or similar molecules [25]. Research undertaken in the related *Lactococcus lactis* has also identified marked downregulation of *glpF2* following osmotic stress [37]. Taken together it would seem that the downregulation of *glpF2* may be a necessary adaptation for M1_UK_ *S. pyogenes *, although it may also confer an as-yet unknown advantage.

The upregulation of SpeA expression by M1_UK_ is clearly of importance to virulence, particularly in interaction with the human host, and those who have not yet mounted an immune response to the secreted toxins of this species. There is good evidence that superantigens such as SpeA undermine development of the adaptive host immune response to *S. pyogenes* through promotion of a dysregulated T cell response associated with B cell death [38, 39]. SpeA has also been shown to promote carriage of *S. pyogenes* in the nasopharynx of transgenic mice [40]. To date, the expression of SpeA has only been measured in broth culture and we do not know if the upregulation in M1_UK_ might differ *in vivo*. Recent epidemiological studies found a high (44%) secondary infection rate in schoolchildren and household contacts of a case of scarlet fever caused by M1_UK_, pointing to a potential transmission advantage compared with other *S. pyogenes* lineages [41]. We identified sublineage-specific altered expression of SpeA, allowing us to highlight the genetic changes likely to account for this. Importantly, the three SNPs identified in the major regulator *rofA* do not alone account for the SpeA phenotype since these SNPs are present in M1_13SNPs_, although we cannot discount a role for these in the wider success of this lineage. While the genetic changes required for increased SpeA expression do not reside in M1_13SNPs_, they do reside in M1_23SNPs_, and strains with reversion of single SNPs pointed to a potential key role for the *ssrA* SNP in SpeA upregulation. The amount of SpeA made by M1_UK_ and M1_23SNPs_ was augmented to the level of M1_global_ *covRS* mutants yet presumably without the fitness burden of *covRS* mutation that might impair pharyngeal carriage [42].

There are a number of limitations to our study. First, investigation of the *gldA* operon is in its early stages; it is possible that the premature stop codon mutation in *gldA* confers an additional phenotype that is not recapitulated by *gldA* gene deletion, while the metabolic pathways that include GldA, MipB and PflD are not fully understood. The roles of GlpF2 and the PTS EII system that is upregulated are also not understood; any role in transfer of DHA, for example, has not been experimentally addressed. The proteomic studies are preliminary and require both validation and repetition using richer media, but have provided a rationale for further study of the role of sugar metabolism in *emm*1 *S. pyogenes *. Finally, the role of specific SNPs would necessarily require experimental proof.

Several European countries are, at the time of writing, affected by epidemic waves of invasive *S. pyogenes* disease, notably in England, where the leading cause of invasive infection is *emm*1, underlining the importance of understanding pathogenicity and transmission [32, 43]. Importantly, however, despite the enhanced production of SpeA by M1_23SNPs_, this intermediate sublineage did not expand in the manner seen for M1_UK_ in England, and was not detected at all in a 2020 systematic evaluation of >300 invasive *emm*1 isolates from England [5]. This suggests that the fitness of M1_UK_ has required the additional acquisition of four further SNPs. These include three non-synonymous SNPs in phosphate transport ATP binding protein, *pstB*; a PTS galactose-specific IIB component gene; a hypothetical protein; as well as the intergenic SNP upstream to *glpF2*. The amount of SpeA produced by M1_UK_ strains remains an order of magnitude lower than the amount produced by the historic *emm*1 strain NCTC8198 that was used for erythrogenic toxin production, and can produce 2000 ng ml^−1^ [44]. Despite this, the new M1_UK_ lineage has outcompeted M1_23SNPs_ and has replaced older strains, suggesting that the added fitness of M1_UK_ may lie beyond the ability to make SpeA.

## Supplementary Data

Supplementary material 1
