# Supplementary material for: Characterization of emergent toxigenic M1UK Streptococcus pyogenes and associated sublineages
Source: Microb Genom. 2023 Apr 24;9(4):mgen000994. doi: 10.1099/mgen.0.000994 (PMC10210942; doi:10.1099/mgen.0.000994)
Supplement: Supplementary material 1 [file mgen-9-994-s001.pdf]

Supplementary file for manuscript: Characterisation of emergent toxigenic $M1_{UK}$ *Streptococcus pyogenes* and associated sublineages

Ho Kwong Li*, Xiangyun Zhi*, Ana Vieira, Harry Whitwell, Amelia Schricker, Elita Jauneikaite, Hanqi Li, Ahmed Yosef, Ivan Andrew, Laurence Game, Claire E. Turner, Theresa Lamagni, Juliana Coelho, Shiranee Sriskandan

CONTENTS

Supplementary Figure S1	Real-time PCR measurement of *glpF2* transcription
Supplementary Figure S2	GldA operon in different species
Supplementary Figure S3	SpeA production by non-invasive *emm*1 S. pyogenes
Supplementary Figure S4	String analysis of proteomic data: $M1_{global}$ vs. $M1_{UK}$ cellular fractions
Supplementary Figure S5	String analysis of proteomic data: four M1 sublineages compared
Supplementary Table S1	Strains used in experimental work
Supplementary Table S2	Strains and WGS used for phylogenetic tree and SpeA quantification
Supplementary Table S3	Chemically-defined medium composition
Supplementary Table S4	Primers used for RT-qPCR

Supplied in Figshare

Excel Suppl. Files	Pairwise proteomic comparisons used for Volcano plots Figures 5 and 6
Numbers Files	Proteomic comparisons for individual strains (5 per group)
	Expt 1. $M1_{global}$ vs. $M1_{UK}$ cellular fractions (5 per group)
	Expt 2. Four M1 sublineages compared (5 per group)

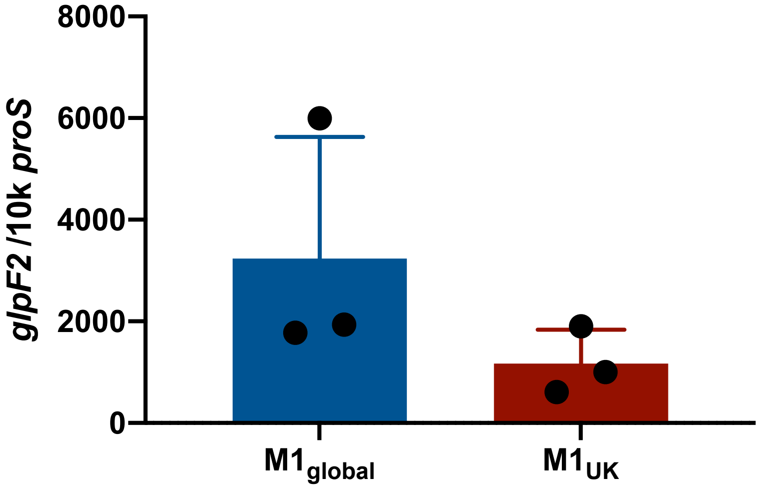

Supplementary Figure S1. Quantitative real-time PCR measurement of *glpF2* transcription in n=3 strains of *S. pyogenes* per lineage. Individual data points represent the average of 3 technical replicates for each strain. Strains used were: $M1_{global}$ (blue bar) (BHS0162; BHS0130; BHS0674) and $M1_{UK}$ (red bar) (BHS0258; BHS0170; BHS0128)

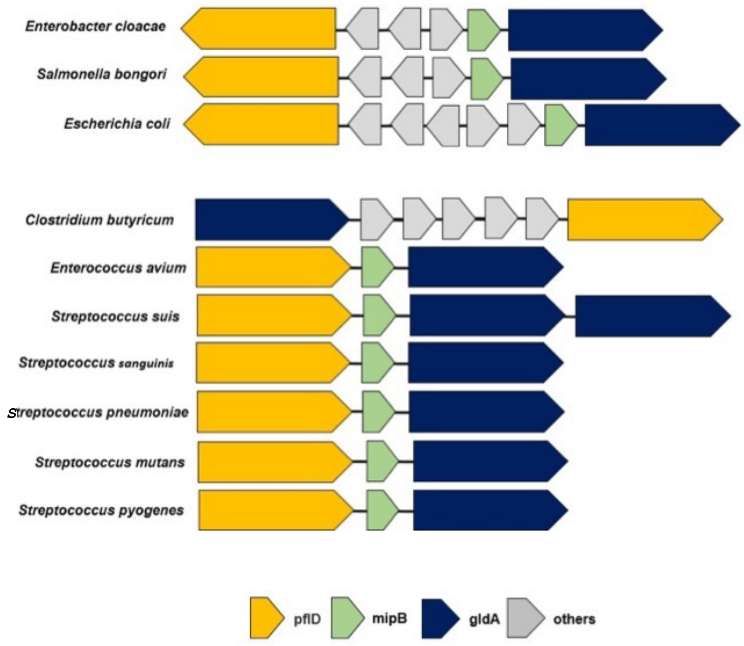

Supplementary Figure S2 GldA operon in different species. Informatic analysis of publicly available genomes from a range of bacterial species demonstrated remarkable conservation of the genes and organisation of this region in all members of the streptococcaciae. Whereas other bacterial species possessed the three genes *mipB* (also annotated as *talC*, for transaldolase), *pflD* and *gldA*, the organisation of genes differed from the sequence observed in streptococci.

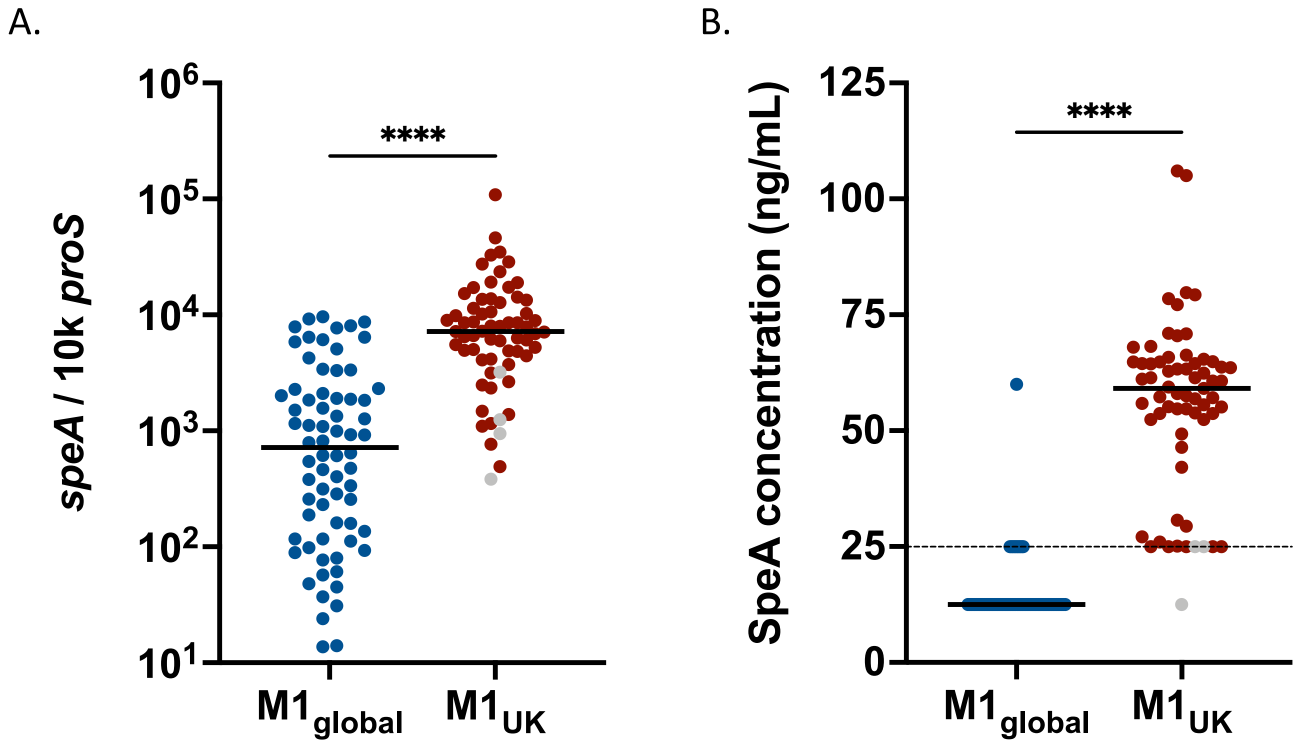

Supplementary Figure S3. Measurement of SpeA production by non-invasive *emm*1 *S. pyogenes* isolates, (A), Quantitative real-time PCR measurement of *speA* transcription in $M1_{global}$ and $M1_{UK}$ strains of *S. pyogenes* per lineage (*previously published in Lynskey N. & Jauneikaite E. et al, Lancet Infect Dis, 2019 Nov; 19(11): 1209-1218, shown for reference only*). (B) Semi quantitative measurement of SpeA expression by western blotting. N=135 strains. Solid line represents the median, dashed line represents the limit of detection. Strains with undetectable SpeA assigned value of 12.5 ng/mL. The two top-producing $M1_{UK}$ strains in panel B represent 2 of 3 strains with mutations in covRS. Grey dots indicate an intermediate sublineage of M1. Unpaired T-test, **** is p<0.0001.

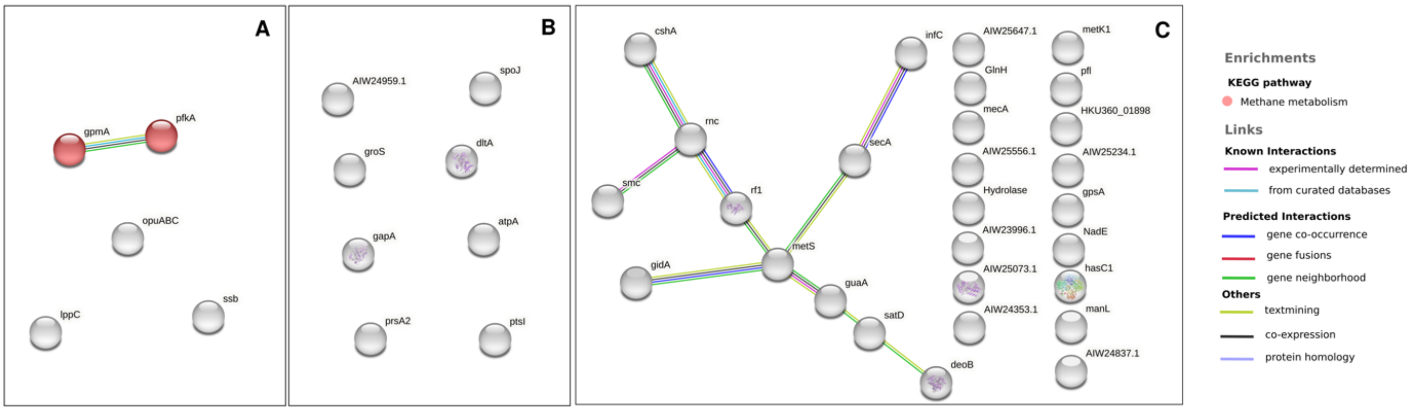

Supplementary Figure S4. Enrichment and protein-protein interactions of differentially expressed proteins (p-value< 0.05) in different bacterial fractions using String database. Coloured lines represent physical associations as described in the legend above. Coloured bubbles represent the type of enrichment observed. Data compare $M1_{UK}$ and $M1_{global}$ Supernatants (A); Cell wall extracts (B); Cytosol extracts (C) obtained from *S. pyogenes* strains cultured in chemically defined medium (CDM). (N=5 different strains per group). Pairwise comparisons are provided in supplementary Excel file.

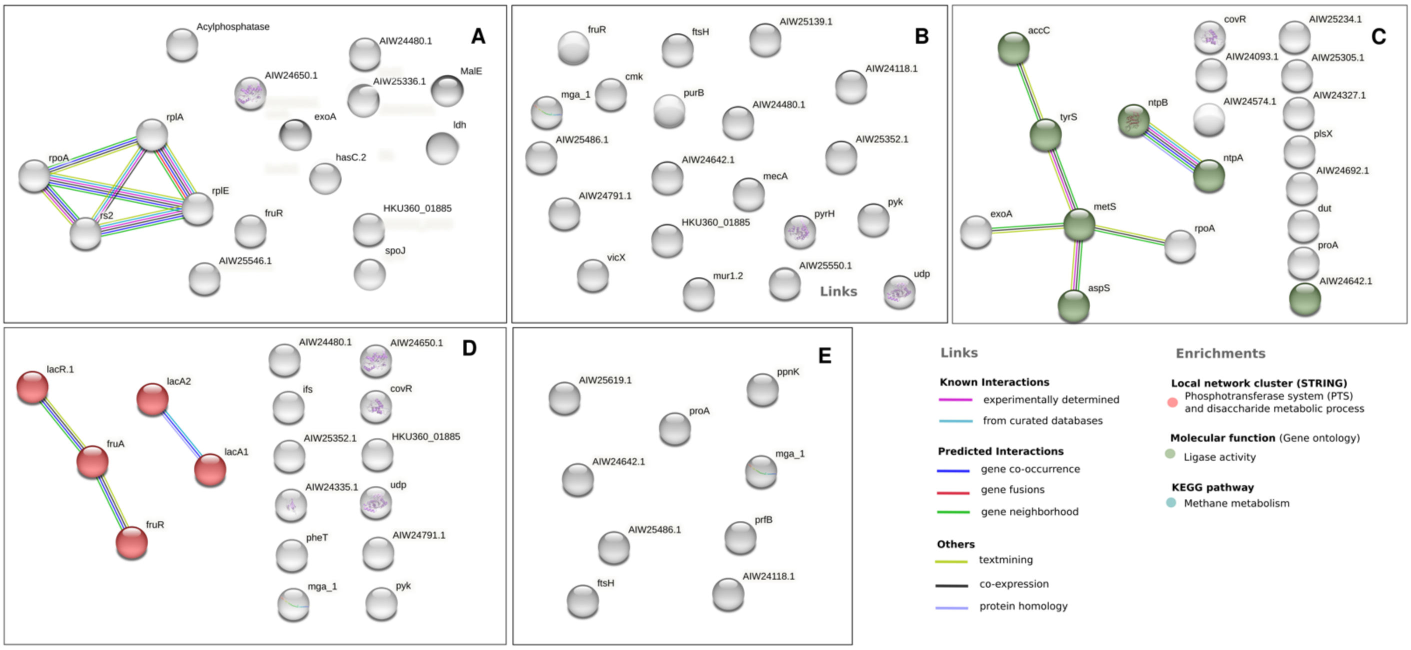

Supplementary Figure S5. Enrichment and protein-protein interactions of differentially expressed proteins (cytosol only, p-value< 0.05) using String database comparing strains from four sublineages $M1_{UK}$; $M1_{23SNPs}$; $M1_{13SNPs}$; and $M1_{global}$ cultured in CDM (N=5 per group). Coloured lines represent physical associations as described in the legend above. Coloured bubbles represent the type of enrichment observed. Associations shown represent: (A) All 4 groups compared, (B) $M1_{global}$ vs $M1_{UK,}$ (C) [$M1_{UK}$+$M1_{23SNPs}$] vs [$M1_{13SNPs}$ + $M1_{global}$], (D) $M1_{global}$ vs all others, (E) $M1_{UK}$ vs all others. Pairwise comparisons are provided in supplementary Excel file.

Supplementary tables

Supplementary Table S1. Bacterial strains used in RNA sequencing and RT-PCR

	**Accession¶**	**Strain identifier**	**Lineage**	**Year**	**RNAseq**	**gldA operon**	**glpF2**
**$M1_{global}$**	ERS1020620	BHS0674	$M1_{global}$	2015	Y	Y	Y
	ERS1020158	BHS0162	$M1_{global}$	2012	Y	Y	Y
	ERS1020095	BHS0130	$M1_{global}$	2011	Y	Y	Y
	ERS1020136	BHS0151 or H1488	$M1_{global}$	2012	Y		
	ERS1020472	BHS0503	$M1_{global}$	2010		Y	
	ERS1020045	BHS0013	$M1_{global}$	2009		Y	
	ERS1020385	BHS0368	$M1_{global}$	2009		Y	
	ERS1020523	BHS0448	$M1_{global}$	2009		Y	
**$M1_{UK}$**	ERS1020174	BHS0170	$M1_{UK}$	2012	Y	Y	Y
	ERS1020090	BHS0128	$M1_{UK}$	2011	Y	Y	Y
	ERS1020341	BHS0258	$M1_{UK}$	2014	Y	Y	Y
	ERS1020603	BHS0581	$M1_{UK}$	2015	Y		
	ERS1020508	BHS0521	$M1_{UK}$	2015		Y	
	ERS1020714	BHS0643	$M1_{UK}$	2015		Y	
	ERS1463088	BHS0762	$M1_{UK}$	2016		Y	

¶Accession numbers relate to genome sequences deposited in the European nucleotide archive

Supplementary Table S2. *S. pyogenes* strains and genome sequences used to create phylogenetic tree (Figure 2) and used to quantify SpeA expression by non-invasive and invasive strains.

ENA¶†	STUDY	DISEASE	LINEAGE	LOCATION	YEAR
ERR2864947	Sharma *et al.*	Unknown	M1global	UK	2017
ERR2864949	Sharma *et al.*	Unknown	M1global	UK	2017
ERR2864951	Sharma *et al.*	Unknown	M1global	UK	2017
ERR2864964	Sharma *et al.*	Unknown	M1global	UK	2017
ERR2864950	Sharma *et al.*	Outbreak	23 SNPs	UK	2017
ERR2864953	Sharma *et al.*	Outbreak	23 SNPs	UK	2017
ERR2864957	Sharma *et al.*	Outbreak	22 SNPs	UK	2017
ERR2864962	Sharma *et al.*	Outbreak	23 SNPs	UK	2017
ERR2864966	Sharma *et al.*	Outbreak	23 SNPs	UK	2017
ERR2864969	Sharma *et al.*	Outbreak	23 SNPs	UK	2017
ERR2864948	Sharma *et al.*	Unknown	M1uk	UK	2017
ERR2864952	Sharma *et al.*	Unknown	M1uk	UK	2017
ERR2864954	Sharma *et al.*	Unknown	M1uk	UK	2017
ERR2864955	Sharma *et al.*	Unknown	M1uk	UK	2017
ERR2864956	Sharma *et al.*	Unknown	M1uk	UK	2017
ERR2864958	Sharma *et al.*	Unknown	M1uk	UK	2017
ERR2864959	Sharma *et al.*	Unknown	M1uk	UK	2017
ERR2864960	Sharma *et al.*	Unknown	M1uk	UK	2017
ERR2864961	Sharma *et al.*	Unknown	M1uk	UK	2017
ERR2864963	Sharma *et al.*	Unknown	M1uk	UK	2017
ERR2864965	Sharma *et al.*	Unknown	M1uk	UK	2017
ERR2864967	Sharma *et al.*	Unknown	M1uk	UK	2017
ERR2864968	Sharma *et al.*	Unknown	M1uk	UK	2017
ERS4267588	This Study	Outbreak	26 SNPs	UK	2018
ERS4267589	This Study	Outbreak	27 SNPs	UK	2018
CP000017.2	Sumby *et al.*	Reference	M1global	x	x
ERS1448799	Kapatai *et al.*	Invasive	13 SNPs	UK	2014
ERS1450651	Kapatai *et al.*	Invasive	13 SNPs	UK	2015
ERS1450822	Kapatai *et al.*	Invasive	13 SNPs	UK	2015
ERS1448193	Kapatai *et al.*	Invasive	23 SNPs	UK	2014
ERS1450815	Kapatai *et al.*	Invasive	23 SNPs	UK	2015
ERS1448173	Kapatai *et al.*	Invasive	M1global	UK	2014
ERS1448879	Kapatai *et al.*	Invasive	M1global	UK	2014
ERS1450607	Kapatai *et al.*	Invasive	M1global	UK	2015
ERS1450839	Kapatai *et al.*	Invasive	M1global	UK	2015
ERS1448481	Kapatai *et al.*	Invasive	M1uk	UK	2014
ERS1449006	Kapatai *et al.*	Invasive	M1uk	UK	2014
ERS1450390	Kapatai *et al.*	Invasive	M1uk	UK	2015
ERS1450879	Kapatai *et al.*	Invasive	M1uk	UK	2015
ERS1594714	Lynskey & Jauneikaite *et al.*	Invasive	13 SNPs	UK	2013
ERS1594852	Lynskey & Jauneikaite *et al.*	Invasive	13 SNPs	UK	2013
ERS1594863	Lynskey & Jauneikaite *et al.*	Invasive	13 SNPs	UK	2013
ERS1594843	Lynskey & Jauneikaite *et al.*	Invasive	13 SNPs	UK	2013
ERS1594990	Lynskey & Jauneikaite *et al.*	Invasive	13 SNPs	UK	2016
ERS1594904	Lynskey & Jauneikaite *et al.*	Invasive	13 SNPs	UK	2016
ERS1594914	Lynskey & Jauneikaite *et al.*	Invasive	13 SNPs	UK	2016
ERS1594824	Lynskey & Jauneikaite *et al.*	Invasive	23 SNPs	UK	2013
ERS1594734	Lynskey & Jauneikaite *et al.*	Invasive	23 SNPs	UK	2013
ERS1594744	Lynskey & Jauneikaite *et al.*	Invasive	23 SNPs	UK	2013
ERS1594757	Lynskey & Jauneikaite *et al.*	Invasive	23 SNPs	UK	2013
ERS1594864	Lynskey & Jauneikaite *et al.*	Invasive	23 SNPs	UK	2013
ERS1594882	Lynskey & Jauneikaite *et al.*	Invasive	23 SNPs	UK	2013
ERS1594950	Lynskey & Jauneikaite *et al.*	Invasive	23 SNPs	UK	2016

ERS1595205	Lynskey & Jauneikaite *et al.*	Invasive	23 SNPs	UK	2016
ERS1594782	Lynskey & Jauneikaite *et al.*	Invasive	M1global	UK	2013
ERS1594798	Lynskey & Jauneikaite *et al.*	Invasive	M1global	UK	2013
ERS1594822	Lynskey & Jauneikaite *et al.*	Invasive	M1global	UK	2013
ERS1594988	Lynskey & Jauneikaite *et al.*	Invasive	M1global	UK	2016
ERS1595222	Lynskey & Jauneikaite *et al.*	Invasive	M1global	UK	2016
ERS1595038	Lynskey & Jauneikaite *et al.*	Invasive	M1global	UK	2016
ERS1594786	Lynskey & Jauneikaite *et al.*	Invasive	M1uk	UK	2013
ERS1594722	Lynskey & Jauneikaite *et al.*	Invasive	M1uk	UK	2013
ERS1594847	Lynskey & Jauneikaite *et al.*	Invasive	M1uk	UK	2013
ERS1594947	Lynskey & Jauneikaite *et al.*	Invasive	M1uk	UK	2016
ERS1595232	Lynskey & Jauneikaite *et al.*	Invasive	M1uk	UK	2016
ERS1595131	Lynskey & Jauneikaite *et al.*	Invasive	M1uk	UK	2016
ERS1020045	Lynskey & Jauneikaite *et al.*	Non invasive	M1global	UK	2009
ERS1020472	Lynskey & Jauneikaite *et al.*	Non invasive	M1global	UK	2010
ERS1020385	Lynskey & Jauneikaite *et al.*	Non invasive	M1global	UK	2009
ERS1020523	Lynskey & Jauneikaite *et al.*	Non invasive	M1global	UK	2009
ERS1020620	Lynskey & Jauneikaite *et al.*	Non invasive	M1global	UK	2015
ERS1463088	Lynskey & Jauneikaite *et al.*	Non invasive	M1uk	UK	2016
ERS1020174	Lynskey & Jauneikaite *et al.*	Non invasive	M1uk	UK	2012
ERS1020714	Lynskey & Jauneikaite *et al.*	Non invasive	M1uk	UK	2015
ERS1020508	Lynskey & Jauneikaite *et al.*	Non invasive	M1uk	UK	2015
ERS1020090	Lynskey & Jauneikaite *et al.*	Non invasive	M1uk	UK	2011
ERS1020076	Lynskey & Jauneikaite *et al.*	Non invasive	M1global	UK	2010
ERS1020092	Lynskey & Jauneikaite *et al.*	Non invasive	M1global	UK	2010
ERS1020107	Lynskey & Jauneikaite *et al.*	Non invasive	M1global	UK	2010
ERS1020141	Lynskey & Jauneikaite *et al.*	Non invasive	M1global	UK	2010
ERS1020171	Lynskey & Jauneikaite *et al.*	Non invasive	13 SNPs	UK	2011
ERS1020034	Lynskey & Jauneikaite *et al.*	Non invasive	M1global	UK	2011
ERS1020036	Lynskey & Jauneikaite *et al.*	Non invasive	M1global	UK	2011
ERS1020046	Lynskey & Jauneikaite *et al.*	Non invasive	M1global	UK	2011
ERS1020050	Lynskey & Jauneikaite *et al.*	Non invasive	13 SNPs	UK	2011
ERS1020056	Lynskey & Jauneikaite *et al.*	Non invasive	M1global	UK	2011
ERS1020079	Lynskey & Jauneikaite *et al.*	Non invasive	M1global	UK	2011
ERS1020095	Lynskey & Jauneikaite *et al.*	Non invasive	M1global	UK	2011
ERS1020102	Lynskey & Jauneikaite *et al.*	Non invasive	M1global	UK	2011
ERS1020104	Lynskey & Jauneikaite *et al.*	Non invasive	M1global	UK	2011
ERS1020128	Lynskey & Jauneikaite *et al.*	Non invasive	M1global	UK	2010
ERS1020136	Lynskey & Jauneikaite *et al.*	Non invasive	M1global	UK	2012
ERS1020152	Lynskey & Jauneikaite *et al.*	Non invasive	M1global	UK	2012
ERS1020156	Lynskey & Jauneikaite *et al.*	Non invasive	23 SNPs	UK	2012
ERS1020158	Lynskey & Jauneikaite *et al.*	Non invasive	M1global	UK	2012
ERS1020162	Lynskey & Jauneikaite *et al.*	Non invasive	M1global	UK	2010
ERS1020199	Lynskey & Jauneikaite *et al.*	Non invasive	M1global	UK	2012
ERS1020207	Lynskey & Jauneikaite *et al.*	Non invasive	M1uk	UK	2012
ERS1020240	Lynskey & Jauneikaite *et al.*	Non invasive	M1uk	UK	2014
ERS1020250	Lynskey & Jauneikaite *et al.*	Non invasive	M1global	UK	2014
ERS1020275	Lynskey & Jauneikaite *et al.*	Non invasive	M1uk	UK	2014
ERS1020277	Lynskey & Jauneikaite *et al.*	Non invasive	M1global	UK	2014
ERS1020341	Lynskey & Jauneikaite *et al.*	Non invasive	M1uk	UK	2014
ERS1020354	Lynskey & Jauneikaite *et al.*	Non invasive	M1global	UK	2014
ERS1020358	Lynskey & Jauneikaite *et al.*	Non invasive	M1uk	UK	2014
ERS1020374	Lynskey & Jauneikaite *et al.*	Non invasive	M1global	UK	2014
ERS1020253	Lynskey & Jauneikaite *et al.*	Non invasive	M1global	UK	2014
ERS1020361	Lynskey & Jauneikaite *et al.*	Non invasive	M1global	UK	2009
ERS1020365	Lynskey & Jauneikaite *et al.*	Non invasive	M1global	UK	2009
ERS1020393	Lynskey & Jauneikaite *et al.*	Non invasive	M1global	UK	2009
ERS1020398	Lynskey & Jauneikaite *et al.*	Non invasive	M1global	UK	2009
ERS1020429	Lynskey & Jauneikaite *et al.*	Non invasive	M1global	UK	2009
ERS1020433	Lynskey & Jauneikaite *et al.*	Non invasive	M1global	UK	2009

ERS1020441	Lynskey & Jauneikaite *et al.*	Non invasive	M1global	UK	2009
ERS1020443	Lynskey & Jauneikaite *et al.*	Non invasive	M1global	UK	2009
ERS1020465	Lynskey & Jauneikaite *et al.*	Non invasive	13 SNPs	UK	2009
ERS1020497	Lynskey & Jauneikaite *et al.*	Non invasive	M1global	UK	2009
ERS1020539	Lynskey & Jauneikaite *et al.*	Non invasive	M1global	UK	2009
ERS1020551	Lynskey & Jauneikaite *et al.*	Non invasive	13 SNPs	UK	2009
ERS1020555	Lynskey & Jauneikaite *et al.*	Non invasive	M1global	UK	2009
ERS1020572	Lynskey & Jauneikaite *et al.*	Non invasive	M1global	UK	2009
ERS1020576	Lynskey & Jauneikaite *et al.*	Non invasive	M1global	UK	2009
ERS1020435	Lynskey & Jauneikaite *et al.*	Non invasive	M1global	UK	2010
ERS1020460	Lynskey & Jauneikaite *et al.*	Non invasive	M1global	UK	2010
ERS1020486	Lynskey & Jauneikaite *et al.*	Non invasive	M1uk	UK	2015
ERS1020488	Lynskey & Jauneikaite *et al.*	Non invasive	M1global	UK	2015
ERS1020502	Lynskey & Jauneikaite *et al.*	Non invasive	M1uk	UK	2015
ERS1020514	Lynskey & Jauneikaite *et al.*	Non invasive	M1global	UK	2015
ERS1020561	Lynskey & Jauneikaite *et al.*	Non invasive	M1global	UK	2015
ERS1020602	Lynskey & Jauneikaite *et al.*	Non invasive	M1global	UK	2015
ERS1020603	Lynskey & Jauneikaite *et al.*	Non invasive	M1uk	UK	2015
ERS1020613	Lynskey & Jauneikaite *et al.*	Non invasive	M1global	UK	2015
ERS1020634	Lynskey & Jauneikaite *et al.*	Non invasive	M1uk	UK	2015
ERS1020638	Lynskey & Jauneikaite *et al.*	Non invasive	M1global	UK	2015
ERS1020646	Lynskey & Jauneikaite *et al.*	Non invasive	M1uk	UK	2015
ERS1020668	Lynskey & Jauneikaite *et al.*	Non invasive	M1uk	UK	2015
ERS1020679	Lynskey & Jauneikaite *et al.*	Non invasive	M1uk	UK	2015
ERS1020688	Lynskey & Jauneikaite *et al.*	Non invasive	M1uk	UK	2015
ERS1020696	Lynskey & Jauneikaite *et al.*	Non invasive	M1global	UK	2015
ERS1020702	Lynskey & Jauneikaite *et al.*	Non invasive	M1uk	UK	2015
ERS1020703	Lynskey & Jauneikaite *et al.*	Non invasive	M1uk	UK	2015
ERS1020705	Lynskey & Jauneikaite *et al.*	Non invasive	M1uk	UK	2015
ERS1020715	Lynskey & Jauneikaite *et al.*	Non invasive	M1global	UK	2015
ERS1020717	Lynskey & Jauneikaite *et al.*	Non invasive	M1uk	UK	2015
ERS1020719	Lynskey & Jauneikaite *et al.*	Non invasive	M1uk	UK	2015
ERS1020732	Lynskey & Jauneikaite *et al.*	Non invasive	M1uk	UK	2015
ERS1020604	Lynskey & Jauneikaite *et al.*	Non invasive	M1uk	UK	2015
ERS1020629	Lynskey & Jauneikaite *et al.*	Non invasive	M1uk	UK	2015
ERS1020631	Lynskey & Jauneikaite *et al.*	Non invasive	M1uk	UK	2015
ERS1020651	Lynskey & Jauneikaite *et al.*	Non invasive	M1uk	UK	2015
ERS1020659	Lynskey & Jauneikaite *et al.*	Non invasive	M1uk	UK	2015
ERS1020667	Lynskey & Jauneikaite *et al.*	Non invasive	M1uk	UK	2015
ERS1020687	Lynskey & Jauneikaite *et al.*	Non invasive	M1uk	UK	2010
ERS1020692	Lynskey & Jauneikaite *et al.*	Non invasive	M1global	UK	2012
ERS1020694	Lynskey & Jauneikaite *et al.*	Non invasive	M1uk	UK	2012
ERS1020701	Lynskey & Jauneikaite *et al.*	Non invasive	M1global	UK	2009
ERS1463073	Lynskey & Jauneikaite *et al.*	Non invasive	M1global	UK	2016
ERS1463078	Lynskey & Jauneikaite *et al.*	Non invasive	M1uk	UK	2016
ERS1463079	Lynskey & Jauneikaite *et al.*	Non invasive	M1global	UK	2016
ERS1463083	Lynskey & Jauneikaite *et al.*	Non invasive	M1uk	UK	2016
ERS1463084	Lynskey & Jauneikaite *et al.*	Non invasive	M1global	UK	2016
ERS1463085	Lynskey & Jauneikaite *et al.*	Non invasive	M1uk	UK	2016
ERS1463086	Lynskey & Jauneikaite *et al.*	Non invasive	M1uk	UK	2016
ERS1463087	Lynskey & Jauneikaite *et al.*	Non invasive	M1uk	UK	2016
ERS1463089	Lynskey & Jauneikaite *et al.*	Non invasive	M1uk	UK	2016
ERS1463090	Lynskey & Jauneikaite *et al.*	Non invasive	M1uk	UK	2016
ERS1463091	Lynskey & Jauneikaite *et al.*	Non invasive	M1uk	UK	2016
ERS1463092	Lynskey & Jauneikaite *et al.*	Non invasive	M1uk	UK	2016
ERS1463093	Lynskey & Jauneikaite *et al.*	Non invasive	M1uk	UK	2016
ERS1463095	Lynskey & Jauneikaite *et al.*	Non invasive	M1global	UK	2016
ERS1463096	Lynskey & Jauneikaite *et al.*	Non invasive	M1uk	UK	2016
ERS1463100	Lynskey & Jauneikaite *et al.*	Non invasive	M1global	UK	2016
ERS1463101	Lynskey & Jauneikaite *et al.*	Non invasive	M1uk	UK	2016

ERS1463102	Lynskey & Jauneikaite *et al.*	Non invasive	M1global	UK	2016
ERS1463104	Lynskey & Jauneikaite *et al.*	Non invasive	M1uk	UK	2016
ERS1463105	Lynskey & Jauneikaite *et al.*	Non invasive	M1global	UK	2016
ERS1463106	Lynskey & Jauneikaite *et al.*	Non invasive	M1global	UK	2016
ERS1463107	Lynskey & Jauneikaite *et al.*	Non invasive	M1global	UK	2016
ERS1463108	Lynskey & Jauneikaite *et al.*	Non invasive	M1global	UK	2016
ERS1463109	Lynskey & Jauneikaite *et al.*	Non invasive	M1global	UK	2016
ERS1463110	Lynskey & Jauneikaite *et al.*	Non invasive	M1global	UK	2016
ERS1463111	Lynskey & Jauneikaite *et al.*	Non invasive	M1uk	UK	2016
ERS1463112	Lynskey & Jauneikaite *et al.*	Non invasive	M1global	UK	2016
ERS1463113	Lynskey & Jauneikaite *et al.*	Non invasive	M1uk	UK	2016
ERS1463114	Lynskey & Jauneikaite *et al.*	Non invasive	M1global	UK	2016
ERS1463115	Lynskey & Jauneikaite *et al.*	Non invasive	M1uk	UK	2016
ERS1463116	Lynskey & Jauneikaite *et al.*	Non invasive	M1uk	UK	2016
ERS1463117	Lynskey & Jauneikaite *et al.*	Non invasive	M1global	UK	2016
ERS1463118	Lynskey & Jauneikaite *et al.*	Non invasive	M1uk	UK	2016
ERS1463119	Lynskey & Jauneikaite *et al.*	Non invasive	M1uk	UK	2016
ERS1463120	Lynskey & Jauneikaite *et al.*	Non invasive	M1global	UK	2016
ERS1463121	Lynskey & Jauneikaite *et al.*	Non invasive	M1uk	UK	2016
ERS1463122	Lynskey & Jauneikaite *et al.*	Non invasive	M1uk	UK	2016
ERS1463123	Lynskey & Jauneikaite *et al.*	Non invasive	M1uk	UK	2016
ERS1463124	Lynskey & Jauneikaite *et al.*	Non invasive	M1uk	UK	2016
ERS1463125	Lynskey & Jauneikaite *et al.*	Non invasive	M1uk	UK	2016
ERS1463126	Lynskey & Jauneikaite *et al.*	Non invasive	M1uk	UK	2016
ERS1463127	Lynskey & Jauneikaite *et al.*	Non invasive	M1uk	UK	2016
ERS1463129	Lynskey & Jauneikaite *et al.*	Non invasive	M1uk	UK	2016
ERS1463130	Lynskey & Jauneikaite *et al.*	Non invasive	M1uk	UK	2016
ERS1463131	Lynskey & Jauneikaite *et al.*	Non invasive	M1uk	UK	2016
ERS1463072	Lynskey & Jauneikaite *et al.*	Non invasive	M1uk	UK	2016
ERS1463099	Lynskey & Jauneikaite *et al.*	Non invasive	M1uk	UK	2016
ERS1463128	Lynskey & Jauneikaite *et al.*	Non invasive	M1uk	UK	2016
ERS1463080	Lynskey & Jauneikaite *et al.*	Non invasive	M1uk	UK	2016
ERS362081	Turner *et al.*	Invasive	M1global	UK	2006
ERS362084	Turner *et al.*	Invasive	M1global	UK	2006
ERS362085	Turner *et al.*	Invasive	M1global	UK	2006
ERS362098	Turner *et al.*	Invasive	M1global	UK	2007
ERS362108	Turner *et al.*	Invasive	M1global	UK	2011
ERS362125	Turner *et al.*	Invasive	M1global	UK	2008
ERS362128	Turner *et al.*	Invasive	M1global	UK	2009
ERS362130	Turner *et al.*	Invasive	M1global	UK	2009
ERS362131	Turner *et al.*	Invasive	M1global	UK	2009
ERS362134	Turner *et al.*	Invasive	M1global	UK	2009
ERS362138	Turner *et al.*	Invasive	M1global	UK	2010
ERS361917	Turner *et al.*	Invasive	M1global	UK	2011
ERS361974	Turner *et al.*	Invasive	M1global	UK	2007
ERS361979	Turner *et al.*	Invasive	M1global	UK	2008
ERS361984	Turner *et al.*	Invasive	M1global	UK	2008
ERS361986	Turner *et al.*	Invasive	M1global	UK	2008
ERS361987	Turner *et al.*	Invasive	M1global	UK	2008
ERS361989	Turner *et al.*	Invasive	M1global	UK	2008
ERS361995	Turner *et al.*	Invasive	M1global	UK	2009
ERS362002	Turner *et al.*	Invasive	M1global	UK	2008
ERS362004	Turner *et al.*	Invasive	M1global	UK	2009
ERS362006	Turner *et al.*	Invasive	M1global	UK	2009
ERS362102	Turner *et al.*	Invasive	19 SNPs	UK	2007
ERS362148	Turner *et al.*	Invasive	M1global	UK	2011
ERS362149	Turner *et al.*	Invasive	M1global	UK	2011
ERS362150	Turner *et al.*	Invasive	M1global	UK	2011
ERS362141	Turner *et al.*	Invasive	M1global	UK	2010
ERS362144	Turner *et al.*	Invasive	M1global	UK	2011

ERS362145	Turner *et al.*	Invasive	M1global	UK	2011
ERS362146	Turner *et al.*	Invasive	M1uk	UK	2011
ERS362147	Turner *et al.*	Invasive	M1uk	UK	2011
ERS362165	Turner *et al.*	Invasive	M1global	UK	2011
ERS362031	Turner *et al.*	Invasive	M1global	UK	2005
ERS362033	Turner *et al.*	Invasive	M1global	UK	2005
ERS362040	Turner *et al.*	Invasive	M1global	UK	2005
ERS362047	Turner *et al.*	Invasive	M1global	UK	2005
ERS362051	Turner *et al.*	Invasive	M1global	UK	2006
ERS362058	Turner *et al.*	Invasive	M1global	UK	2006
ERS362063	Turner *et al.*	Invasive	M1global	UK	2006
ERS362066	Turner *et al.*	Invasive	M1global	UK	2006
ERS362067	Turner *et al.*	Invasive	M1global	UK	2006
ERS362071	Turner *et al.*	Invasive	13 SNPs	UK	2006
ERS362110	Turner *et al.*	Invasive	M1global	UK	2003
ERS362122	Turner *et al.*	Invasive	M1global	UK	2005
ERS362029	Turner *et al.*	Invasive	M1global	UK	2005
ERS361855	Turner *et al.*	Invasive	M1global	UK	2001
ERS361867	Turner *et al.*	Invasive	M1global	UK	2002
ERS361913	Turner *et al.*	Invasive	M1global	UK	2004
ERS361937	Turner *et al.*	Invasive	M1global	UK	2004
ERS361941	Turner *et al.*	Invasive	M1global	UK	2004
ERS361950	Turner *et al.*	Invasive	M1global	UK	2004
ERS361954	Turner *et al.*	Invasive	M1global	UK	2004
ERS361960	Turner *et al.*	Invasive	13 SNPs	UK	2005
ERS361966	Turner *et al.*	Invasive	M1global	UK	2004
ERS361929	Turner *et al.*	Invasive	M1global	UK	2004
ERS361826	Turner *et al.*	Invasive	M1global	UK	2001
ERS361827	Turner *et al.*	Invasive	M1global	UK	2001
ERS361841	Turner *et al.*	Invasive	M1global	UK	2001
ERS361865	Turner *et al.*	Invasive	M1global	UK	2002
ERS362030	Turner *et al.*	Invasive	M1global	UK	2005
ERS362032	Turner *et al.*	Invasive	M1global	UK	2005
ERS362049	Turner *et al.*	Invasive	M1global	UK	2005
ERS362078	Turner *et al.*	Invasive	M1global	UK	2006
ERS379360	Turner *et al.*	Invasive	M1global	UK	2006

¶Non-invasive strains that were used in SpeA quantification (Supplementary figure 2) are highlighted by grey shading.

†Invasive strains representing four sublineages used for SpeA quantification are highlighted in blue shading

Supplementary Table S3. Components of chemically defined media (CDM) –

COMPONENT¶	CONSTITUENT	STOCK CONC	VOLUME IN 500mL CDM
Water	n/a	n/a	350
Iron	Fe(NO3)3.9H2O - 50mg	1000X	0.5
	F2SO4.7H2O - 250mg		
	made up to50mL ddH2O		
Phosphate	K2HPO4 - 5g	25X	20
	KH2PO4 - 25g		
	NaH2PO4.H2O - 79.9g		
	Na2HPO4.7H2O - 346.8g		
	made up to 1L ddH2O		
MgSO4.7H2O	MgSO4.7H2O - 87.5g	500X	1
	made up to 250mL ddH2O		
MnSO4.H2O	MnSO4.H2O - 1.25g	1000X	0.5
	made up to 250mL ddH2O		
Sodium acetate	NaC2H3O2.3H2O - 112.5g	50X	10
	made up to 500mL ddH2O		
Calcium chloride	CaCl2.2H2O - 338mg	1000X	0.5
	made up to 50mL ddH2O		
Sodium bicarbonate	NaHCO3 - 50g	20X	25
	made up to 1L dd H2O		
L-Cysteine HCl	Cysteine HCl - 16.25mg	500X	1
	made up to 50mL ddH2O		
Bases	2N HCl - 50mL	100X	5
	adenine - 0.5g		
	guanine hydrochloride - 0.5g		
	uracil - 0.5g		
	ddH2O - 200ml		
Vitamins	p-aminobenzoic acid - 10mg	1000X	0.5
	biotin - 10mg		
	folic acid - 40mg		
	nicotinamide - 50mg		
	b-nicotinamide adenine dinucleotide phosphate - 125mg		
	pantothenate calcium salt - 100mg		
	pyridoxal - 50mg		
	pyridoxamine dihydrochloride - 50mg		
	riboflavin - 100mg		
	thiamine hydrochloride - 50mg		
	vitamin B12 - 5mg		
	dissolve in 50mL ddH2O		
Amino acids	DL- alanine - 2.5g	50X	10

	L-arginine - 2.5g		
	L-aspartic acid - 2.5g		
	L-asparagine - 2.5g		
	L-cystine 1.25g		
	L-glutamic acid - 2.5g		
	L-glutamine - 5.0g		
	Glycine - 2.5g		
	L-histidine - 2.5g		
	L-isoleucine - 2.5g		
	L-leucine - 2.5g		
	L-lysine - 2.5g		
	L-methionine - 2.5g		
	L-phenylalanine - 2.5g		
	L-proline - 2.5g		
	hydroxy-L-proline - 2.5g		
	L-serine - 2.5g		
	L-threonine - 5.0g		
	L-tryptophan - 2.5g		
	L-tyrosine - 2.5g		
	L-valine - 2.5g		
	made up to 500mL ddH2O		
Carbon source	e.g., glucose	n/a	5g

¶adapted from van de Rijn and Kessler, *Infect Immun*, 27(2):444

Supplementary Table S4. Primers used for RT-qPCR

GENE	PRIMER	SEQUENCE	PRODUCT (bp)
gldA	gldA_RTF	GCCTCAGATAATGAAATCAGCC	121
	gldA_RTR	CAAGTAAGTCAGCGATAGCC	
mipB	mipB_RTF	TAGGAGCACAAGCCATCAC	100
	mipB_RTR	TCCCAATCCTTGCCAAAATC	
pflD	pfld_RTF	CAAAAACGGCTAAACCAGAAC	118
	pfld_RTR	ATGAACCAGACAGATTGCAC	
ptsIIc	PTS_RTF	GCAAAACATCATCAAGCCAATC	121
	PTS_RTR	CCCAGCAATCAGGAAAAGAC	
glpF2	glpF_RTF	GCTATGGCTTAGGAGTTATG	120
	glpF_RTR	AGACGTGAGCCCATGGGAAC	
speA	speA_Fwd	GAGGGGTAACAAATCATGAAGG	94
	speA_Rv	TCAAATGATAGGCTTTGGATACC	
proS	proS_Fwd	TGAGTTTATTATGAAAGACGGCTATAGTTTC	93
	proS_Rv	AATAGCTTCGTAAGCTTGACGATAATC	
